# The *Calicophoron daubneyi* genome provides new insight into mechanisms of feeding, eggshell synthesis and parasite-microbe interactions

**DOI:** 10.1186/s12915-025-02114-0

**Published:** 2025-01-13

**Authors:** Shauna M. Clancy, Mark Whitehead, Nicola A. M. Oliver, Kathryn M. Huson, Jake Kyle, Daniel Demartini, Allister Irvine, Fernanda Godoy Santos, Paul-Emile Kajugu, Robert E. B. Hanna, Sharon A. Huws, Russell M. Morphew, J. Herbert Waite, Sam Haldenby, Mark W. Robinson

**Affiliations:** 1 School of Biological Sciences, Queen’s University Belfast, 19 Chlorine Gardens, Belfast, Northern Ireland UK; 2 Centre for Genomic Research, University of Liverpool, Liverpool, UK; 3 Department of Chemistry & Biochemistry, University of California Santa Barbara, Santa Barbara, CA 93106 USA; 4 Agrifood & Biosciences Institute, Belfast, UK; 5 Department of Life Sciences, Aberystwyth University, Aberystwyth, Wales UK

**Keywords:** *Calicophoron daubneyi*, Rumen fluke, Paramphistome, Trematode, Genome, Eggshell, Antimicrobial, Peptidoglycan-recognition protein

## Abstract

**Background:**

The rumen fluke, *Calicophoron daubneyi*, is the major paramphistome species infecting ruminants within Europe. Adult flukes reside within the rumen where they are in direct contact with a unique collection of microorganisms. Here, we report a 1.76-Gb draft genome for *C. daubneyi*, the first for any paramphistome species.

**Results:**

Several gene families have undergone specific expansion in *C. daubneyi*, including the peptidoglycan-recognition proteins (PGRPs) and DM9 domain-containing proteins, which function as pattern-recognition receptors, as well as the saposin-like proteins with putative antibacterial properties, and are upregulated upon arrival of the fluke in the microbe-rich rumen. We describe the first characterisation of a helminth PGRP and show that a recombinant *C. daubneyi* PGRP binds to the surface of bacteria, including obligate anaerobes from the rumen, via specific interaction with cell wall peptidoglycan. We reveal that *C. daubneyi* eggshell proteins lack L-DOPA typically required for eggshell crosslinking in trematodes and propose that *C. daubneyi* employs atypical eggshell crosslinking chemistry that produces eggs with greater stability. Finally, although extracellular digestion of rumen ciliates occurs within the *C. daubneyi* gut, unique ultrastructural and biochemical adaptations of the gastrodermal cells suggest that adult flukes also acquire nutrients via uptake of volatile fatty acids from rumen fluid.

**Conclusions:**

Our findings suggest that unique selective pressures, associated with inhabiting a host environment so rich in microbial diversity, have driven the evolution of molecular and morphological adaptations that enable *C. daubneyi* to defend itself against microorganisms, feed and reproduce within the rumen.

**Supplementary Information:**

The online version contains supplementary material available at 10.1186/s12915-025-02114-0.

## Background

A major constraint to the livestock industry is the variety of helminth (worm) species that can infect animals and inflict significant losses in terms of feed intake and conversion efficiency, milk and meat yields, wool production and fertility, etc. Helminths account for > 55% of all farm animal disease which is reflected in a global market of $1.9 billion for veterinary drugs with activity against endoparasites [1, 2]. Whilst several helminth species are endemic in the UK, the prevalence of the rumen fluke, *Calicophoron daubneyi*, in particular has increased considerably in the last 10–15 years [3].

*C. daubneyi* is a member of the family paramphistomidae, a group of flukes generally found in the forestomachs of ruminants, that exhibit a conical shape quite unlike the flattened morphology of other trematodes [4]. Infection occurs when grazing livestock ingest metacercariae that are found encysted on vegetation. Once in the duodenum, newly excysted juvenile (NEJ) flukes emerge and migrate into the intestinal submucosa where they can cause considerable pathology if present in large numbers [5]. If large numbers of metacercariae are ingested (e.g. > 5000 cysts per day over a number of weeks), this can lead to a massive build-up of NEJs in the duodenum which results in acute, clinical paramphistomosis [6]. This is characterised by haemorrhage of damaged regions of the small intestine, causing significant blood loss and hypoalbuminaemia whilst severe cases can be exacerbated by secondary bacterial infection and may be fatal [7, 8].

After feeding on host tissue in the duodenum, the immature flukes begin their migration upwards along the digestive tract to the rumen where they attach to the papillae via their muscular posterior acetabula [9] and begin to produce eggs at around 8–10 weeks post-infection [10]. The mechanism by which mature rumen fluke acquire nutrition from their host remains unclear but may be linked to the differing pathogenicity displayed by different paramphistome species: chronic paramphistomosis caused by mature rumen flukes is considered a production-limiting disease in many tropical and sub-tropical regions where species such as *Paramphistomum cervi* dominate [11, 12]. In contrast, chronic infection due to *C. daubneyi*, the predominant species in Europe [13–15], is not currently associated with clinical disease or impacts on production [16].

The attachment of *C. daubneyi* to the rumen epithelium brings the parasite into intimate contact with host tissues but also with the plethora of anaerobic bacteria, protozoa, fungi and methanogenic archaea that constitute the rumen microbiome, residing in the main fermentative compartment of the forestomach. These microbes play a crucial role in breaking down indigestible plant-based material, converting complex carbohydrates into volatile fatty acids that serve as a primary source of energy for the host. However, as a by-product, hydrogen is released and utilised by rumen archaea as an electron donor to reduce carbon dioxide producing methane which is a major greenhouse gas (GHG) released from ruminants [17, 18]. The discovery that extracellular vesicles (EVs) secreted by adult *C. daubneyi* can alter rumen bacterial species diversity via direct antimicrobial activity [19] demonstrates that paramphistomes can indeed interact with/modulate the rumen microbiome and perhaps utilise them as a food source.

Although there are over 70 recognised species of rumen fluke [20], nucleotide sequence data that would facilitate molecular-level understanding of their feeding mechanisms and host-/microbiome interactions are lacking. To date, transcriptome datasets have only been generated for *P. cervi* (adults; [21]) and *C. daubneyi* (4 intra-mammalian life-cycle stages; [22, 23]) whilst no genome sequence information exists for any paramphistome species. Here, using PacBio long-range sequencing, we present a high-quality genome assembly for *C. daubneyi* and identify key gene families that have undergone specific expansion in this species. We propose that, together with unique morphological adaptations, the molecules encoded by these genes, confer unique functionality for feeding, reproduction and microbial recognition/defence that allow *C. daubneyi* to survive in a hostile and microorganism-rich host environment. These data will have far-reaching implications for our understanding of host-helminth-microbiome interactions and how these may influence enteric GHG emissions, particularly methane, by livestock [24].

## Results

### The *C. daubneyi* genome assembly

The summary statistics of the *C. daubneyi* genome assembly are shown in Table 1. The final *C. daubneyi* assembly comprised a 1.76-Gb genome sequence which is considerably larger than those of other trematodes such as *C. sinensis* (0.54 Gb), *O. viverrini* (0.60 Gb), *P. westermani* (0.92 Gb) and *F. hepatica* (1.14 / 1.27 Gb) [25–29]. The GC content was 43.14% which was comparable to other trematodes. The completeness of the assembly was assessed by BUSCO, using eukaryote lineage data, and revealed that 78.1% completeness and 2.0% duplication level were achieved. This suggests that the *C. daubneyi* genome is more complete than those described for related species; e.g. *C. sinensis* (70.2–73.2% complete; 0.7–1.3% duplicated), *O. viverrini* (73.1% complete; 1.3% duplicated), *P. westermani* (68.6–70.1% complete; 0.7–1.9% duplicated) and *F. hepatica* (66.5–71.1% complete; 0.9–1.7% duplicated) [30].

**Table 1:** Summary characteristics of the *C. daubneyi* draft genome

Parameter	Value
***Assembly***	
Assembly size (Gb)	1.761
Number of contigs	1047
N50	14,590,010
L50	34
N90	2,346,151
L90	145
GC content (%)	43.14
Repetitive content (%)	36.3
***Annotation***	
Protein encoding genes	17,639
Average gene length (bp)	18,681
Transcripts	19,329
Average exon length (bp)	257.2
Average protein length (AAs)	578.14
BUSCO – complete (%)	78.1 (genome); 93.7 (proteins)
BUSCO – fragmented (%)	10.2 (genome); 2.3 (proteins)
BUSCO – missing (%)	11.7 (genome); 4.0 (proteins)

A considerable proportion of repetitive content (36.3%) was identified in the *C. daubneyi* genome which is average when compared to the levels observed in other trematode species where repeating content ranges from 28.9% in *C. sinensis* to 57.1% in *F. hepatica* [25, 27]. A total of 17,639 protein-encoding genes (and 19,329 putative transcript isoforms) were predicted from the *C. daubneyi* genome based on previously published RNA evidence [23]. Analysis of the gene models using the Funannotate annotate module resulted in 18,712 EggNog mapper annotations, 15,182 PFAM annotations and 502 MEROPS annotations. To assess the completeness of the resulting gene predictions, the predicted protein sequences were submitted to BUSCO to identify single-copy core orthologs. Based on this analysis, the predicted proteome of *C. daubneyi* was estimated to be 93.7% complete. Whilst this is higher than the BUSCO genome score, it may reflect a greater gene prediction accuracy when using proteins on account of redundancy and more conserved amino acid sequence.

### Phylogenetic and biochemical relationships within the platyhelminthes

**Fig. 1:**
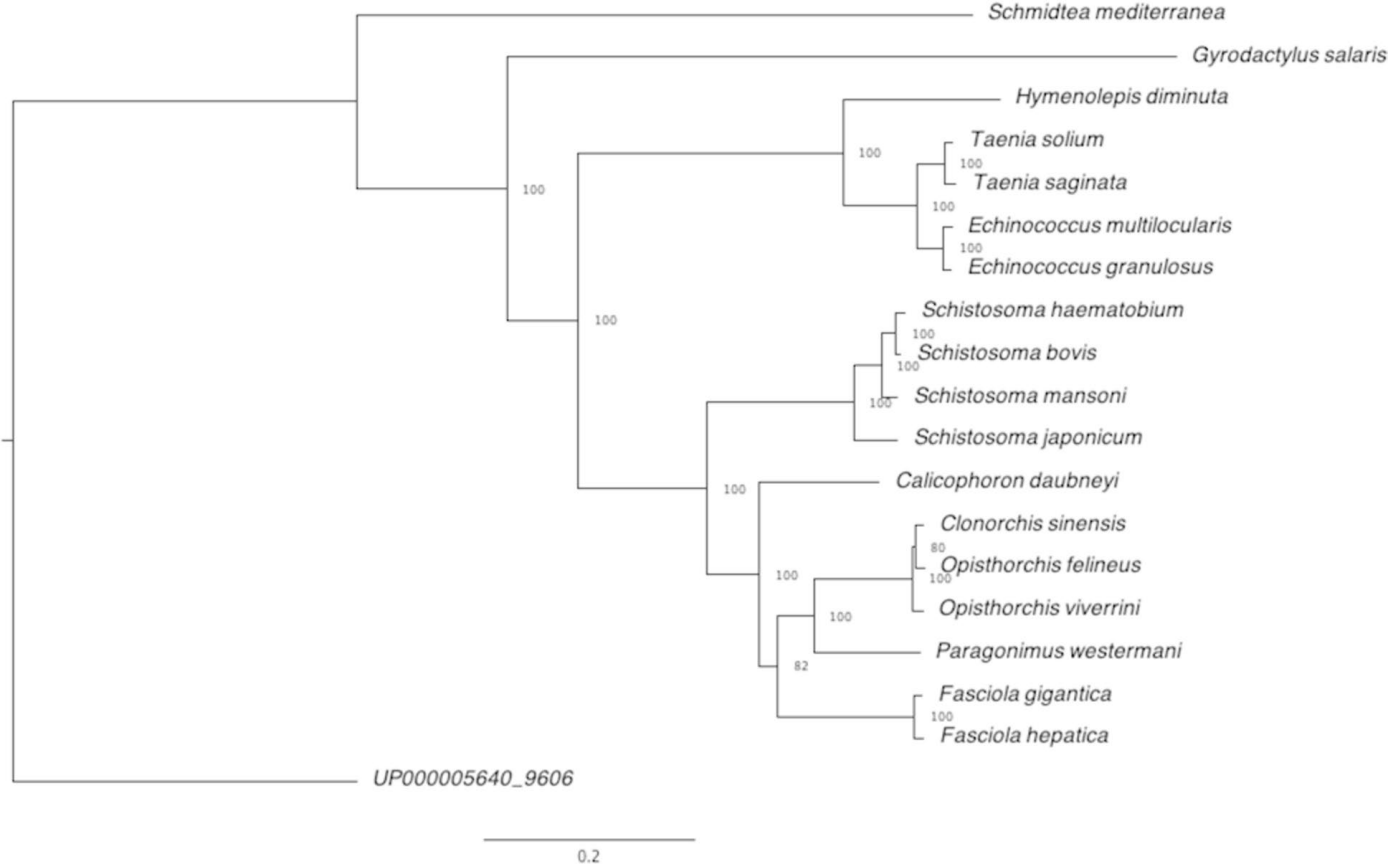
Phylogenetic tree of 18 platyhelminth species based on 180 shared single-copy orthologues using the using the LG + I + G4 model amino acid substitution model. Human sequences were used as an outgroup. Values represent bootstrap support as a percentage

Phylogenetic analysis was performed using 180 shared single-copy orthologues from *C. daubneyi* and 16 other trematode/cestode/monogenean species and the free-living turbellarian *Schmidtea mediterranea* selected to represent the main phylogenetic groups and with protein BUSCO scores > 80%*.* Human sequences were used as an outgroup. Briefly, the longest isoform protein sets were concatenated into a single alignment and submitted to modeltest-ng to obtain the best-fit model for amino acid substitution. The resulting model (LG + I + G4) was provided to raxml-ng along with the concatenated single alignment to generate the final alignment. In the resulting phylogenetic tree, *S. mediterranea* and monogenean *G. salaris* both segregated into monophyletic groups, albeit represented here with a single species, leaving the cestoda and trematoda as sister-groups (Fig. 1). Within the trematoda clade, *C. daubneyi* segregated from the non-schistosome trematodes (likely representing a paramphistomidae monophyletic group) after the split from the schistosomes.

**Fig. 2:**
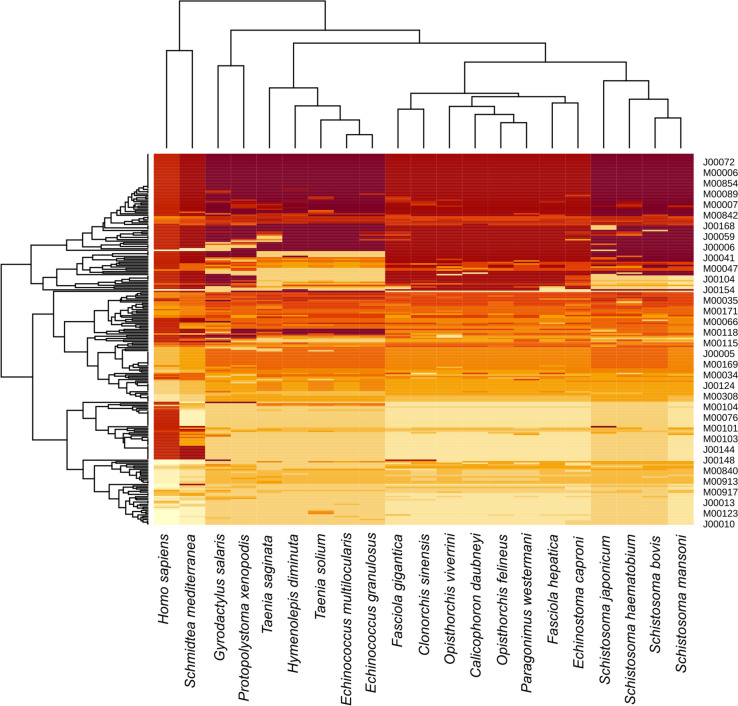
Conservation of metabolic pathways across 20 platyhelminth species represented as a heatmap based on the presence/absence of KEGG modules. Modules with no representation across all species were removed. Darker colours represent higher proportions of genes present within the KEGG module, whilst lighter colours represent absent modules

In the context of understanding how rumen fluke have adapted to survive in the rumen, and how they acquire nutrition, metabolic pathways were reconstructed for *C. daubneyi* and 19 other platyhelminth species based on the proportion of complete KEGG modules. Using this approach, the free-living *S. mediterranea* segregated from the parasitic species, whilst the monogeneans formed a sister group with the trematodes. Overall, the biochemical pathways used by *C. daubneyi* were similar to other non-schistosome species, particularly *O. viverrini*, *O. felineus* and *P. westermani* (Fig. 2; Additional file 1).

### A number of gene families have undergone expansion in *C. daubneyi*

Although the *C. daubneyi* genome showed a similar level of conservation of biochemical (KEGG) pathways as other trematodes, a number of gene families have undergone significant expansion/contraction. Initial analysis using Cafe5 revealed 1800 significantly changed orthogroups (out of ~ 18,000 orthogroups containing at least 2 genes; *p* < 0.05). In contrast, GLM analysis identified 137 and 38 significantly altered orthogroups, using standard GLM parameters or a GLM/zero-inflated model, respectively. Thirteen orthogroup expansions shared between the Cafe5 analysis and the GLM-zero-inflated model are shown in Table 2.

**Table 2:** Expanded gene families in *C. daubneyi*

OG	Function	*C. daubneyi* gene count	Helminth gene count^a^	CAFÉ *p* value	GLM *p* value
OG0000234	LicD family	19	2.3	< 0.001	< 0.001
OG0000284	Diacylglycerol O-acyltransferase 2	16	1.8	< 0.001	< 0.001
OG0000541	Alpha-(1,6)-fucosyltransferase	11	1.7	< 0.001	< 0.001
OG0000554	Calmodulin-like protein	14	1.5	< 0.001	< 0.001
OG0000797	Enoyl-Acyl carrier protein reductase	11	1.0	< 0.001	0.001
OG0000828	Myoglobin	13	1.2	0.04	< 0.001
OG0001071	Multiple inositol polyphosphate phosphatase	6	1.4	0.002	0.001
OG0001742	Uncharacterised protein	10	0.8	< 0.001	< 0.001
OG0001766	Uncharacterised protein	7	1.0	0.007	< 0.001
OG0002059	ADP-ribose glycohydrolase	6	1.0	0.013	< 0.001
OG0002976	Peptidoglycan-recognition protein	11	0.4	< 0.001	< 0.001
OG0005388	Von Willebrand factor A-like	6	0.7	< 0.001	< 0.001
OG0005485	Nicotinamide riboside kinase	5	0.5	0.049	< 0.001

Orthogroups (OG) were identified using Orthofinder and changes in gene family members were assessed using CAFÉ (v5) and GLM analysis using Maaslin2. A final set of 13 OGs, representing 135 genes, were significantly expanded in both analyses

^a^The mean gene count of each OG from 17 platyhelminth species (representing the major taxonomic groups) are shown for comparison

These included OG0000234, comprised of 19 genes encoding members of the LicD transferase family (IPR007074) which are involved with lipopolysaccharide modification in bacteria [31]; OG0000284 representing 16 genes encoding diacylglycerol O-acyltransferases (IPR007130) that participate in triacylglycerol synthesis [32]; OG0000541, comprised of 11 genes encoding alpha-(1,6)-fucosyltransferases (IPR045573) which participate in core fucosylation of *N*-linked glycans [33]; OG0000554 representing 14 genes encoding calmodulin-like proteins (IPR002048) which contribute to calcium signalling pathways and regulate growth in *F. hepatica* [34]; OG0000797, comprising 11 genes encoding enoyl-Acyl carrier protein reductases (IPR002347) which participate in the fatty acid elongation cycle [35]; OG0000828, representing 13 genes encoding the oxygen carrier myoglobin (IPR011406) [36]; OG0001071, comprising 6 genes encoding multiple inositol polyphosphate phosphatases (IPR) which may regulate cellular phosphate levels [37]; OG0001742 and OG0001766, representing 10 and 7 genes encoding uncharacterised proteins respectively; OG0002059, comprising 6 genes encoding ADP-ribose glycohydrolase (IPR005502) which perform ADP-ribosylations [38]; OG0002976, representing 11 genes encoding peptidoglycan-recognition proteins (PGRPs; IPR015510) which are pattern-recognition receptors (PRRs) with important roles in innate immunity [39]; OG0005388, containing 6 genes encoding Von Willebrand factor A-like proteins (IPR013694) which may inhibit plasma proteases or help stabilise the extracellular matrix [40] and OG0005485, representing 5 genes encoding nicotinamide riboside kinases (IPR027417) which participate in cofactor biosynthesis [41].

### *C. daubneyi* has an expanded repertoire of pattern-recognition receptors and putative antimicrobial molecules

**Fig. 3:**
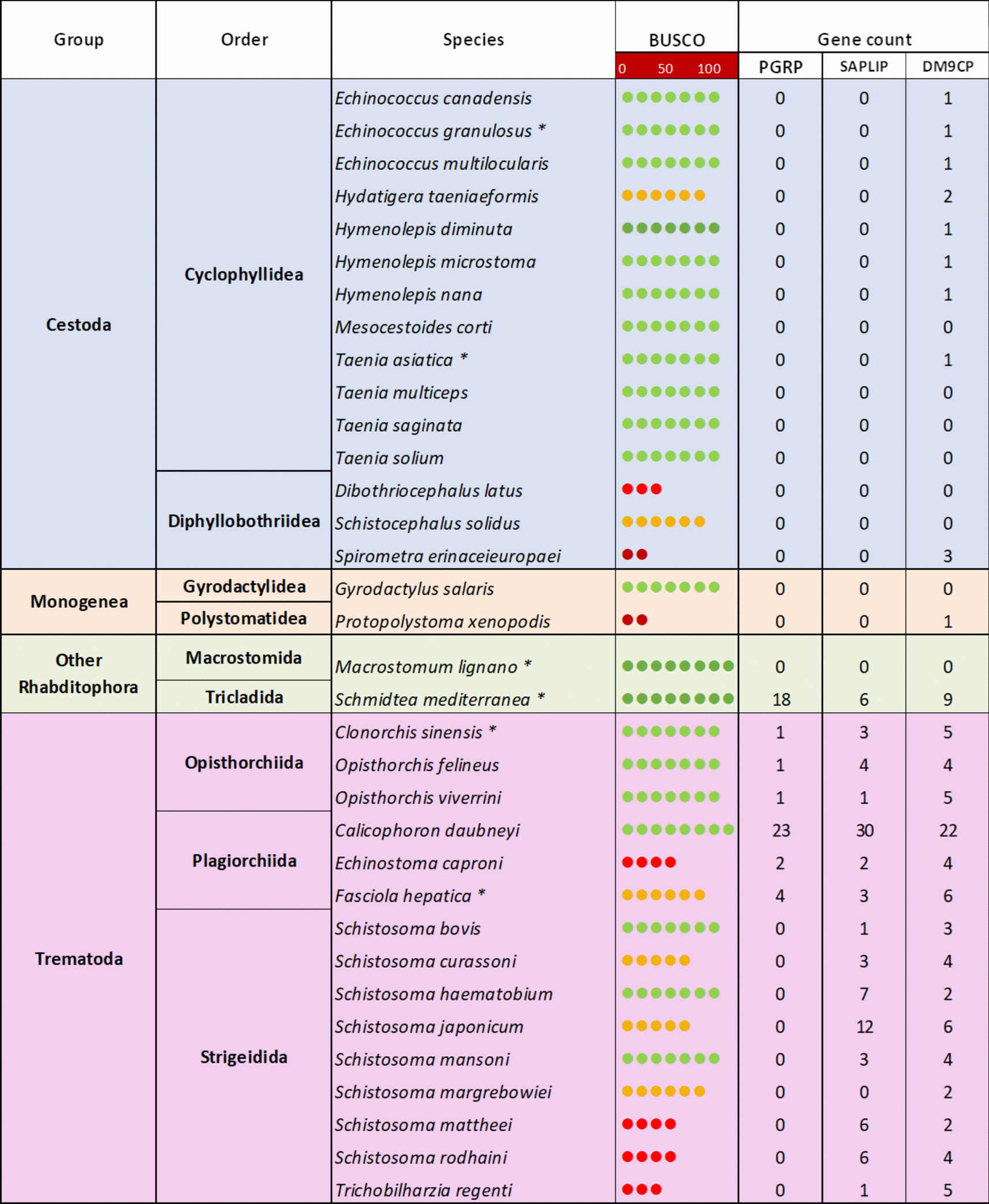
Relative abundance of pattern recognition receptor/antimicrobial genes across 34 representative platyhelminth genomes. Gene counts were calculated following BLASTP analysis (1 × 10^−6^ e-value cut-off). BUSCO scores were obtained from WormBase ParaSite V14 [30]. *multiple genome assemblies were used

The expansion of the PGRP gene family in the rumen fluke genome was of particular interest given the intimate contact of *C. daubneyi* with the array of bacteria in host rumen fluid. Accordingly, other PRR genes or those with putative antibacterial activity, whose products were previously identified within the secretome of *C. daubneyi* [23] were identified via BLAST analysis (Fig. 3). Using this approach, a total of 23 sequences encoding PGRPs were confirmed (representing 18 genes and 5 isoforms) (Additional file 2). Phylogenetic analysis showed that these sequences separated into 4 distinct clades (Additional file 3). The majority (15 genes) represented short-form PGRPs, encoding proteins of ~ 200 amino acids in length (~ 20 kDa).

These contained the characteristic PGRP/*N*-acetylmuromoyl-L-alanine amidase domain (IPR015510/IPR002502), within which conserved residues comprising Zn^2+^-binding (His^55^, His^164^ and His^172^), substrate binding (His^56^, Thr^57^, Asp^86^, Tyr^90^, Arg^104^, Ala^111^, His^112^, Asn^117^, His^164^, Thr^170^, Asn^171^ and Cys^172^) and amidase catalytic sites (His^55^, Tyr^90^, His^164^, Thr^170^, Cys^172^) were found. The remaining 8 genes encoded long form PGRPs ~ 700–1200 amino acids in length (~ 86–138 kDa) which contained a single ~ 22 amino acid predicted transmembrane helix downstream of the PGRP/*N*-acetylmuromoyl-L-alanine amidase domain. Furthermore, 13 *C. daubneyi* PGRPs possessed a predicted N-terminal signal peptide for secretion whilst 10 did not.

DM9 domain-containing proteins (DM9CPs) are a recently described family of invertebrate PRRs [42]. BLAST analysis identified a total of 22 sequences (representing 20 genes and 2 isoforms) encoding DM9CPs in the *C. daubneyi* genome. Phylogenetic analysis showed that these sequences segregated into 4 distinct clades (Additional file 3). The largest protein was 399 amino acids in length (~ 44 kDa) and contained 2 DM9 repeat domains (IPR006616) whilst the majority were around 150–230 amino acids (~ 16–25 kDa) and contained a single DM9 repeat domain. None of the encoded *C. daubneyi* DM9CPs contained a predicted N-terminal signal peptide.

Saposin-like proteins (SAPLIPs) are members of the amoebapore superfamily of lipid-interacting molecules with antibacterial activities [43]. Focusing on proteins only bearing saposin B type domains (IPR008139; typical of SAPLIPs involved in defence), we identified 30 sequences encoding SAPLIPs (representing 26 genes and 4 isoforms) in the *C. daubneyi* genome. Ten of these showed particular sequence identity with NK lysins (antibacterial proteins that are also amoebapore superfamily members) following reciprocal BLAST analysis. Five major SAPLIP clades were evident following phylogenetic analysis of the amino acid sequences (Additional file 3). Whilst the majority of the *C. daubneyi* SAPLIP genes encoded proteins of ~ 100 amino acids in length (typically ~ 12 kDa) bearing a single saposin B type domain, one 193 amino acid protein (~ 21 kDa) possessed 2 saposin B type domains whilst 2 SAPLIPs (254 and 441 amino acids in length; 28 and 50 kDa, respectively) contained 3 saposin B domains. All but two of the *C. daubneyi* SAPLIPs contained a predicted N-terminal signal peptide.

BLAST analysis of 33 platyhelminth genomes representing the major taxonomic groups, and different niches with the mammalian host, suggests that all three gene families (i.e. PGRPs, DM9CPs and SAPLIPs) have specifically expanded in *C. daubneyi*. For example, the PGRPs were absent from the cestodes and monogeneans and, with the exception of the free-living planarian *S. mediterranea*, were restricted to the non-schistosome trematodes with 23 PGRPs identified in *C. daubneyi*, 4 in *F. hepatica*, 2 in *E. caproni* and 1 in *O. viverrini*, *O. felineus* and *C. sinensis* (Fig. 3). Whilst the DM9CPs displayed a wider taxonomic distribution, the parasitic trematodes possessed 2–6 putative DM9CPs whilst 22 were identified in *C. daubneyi*. Similarly, *C. daubneyi* has at least twice as many SAPLIP genes as the other trematodes for which 2–12 putative SAPLIPs were identified. Although the quality of the various genome assemblies may influence the number of genes reported by homology searches, it is unlikely that this alone accounts for the difference in gene counts between *C. daubneyi* and the other platyhelminth species since species with high BUSCO scores consistently yielded few BLAST hits (Fig. 3).

**Fig. 4:**
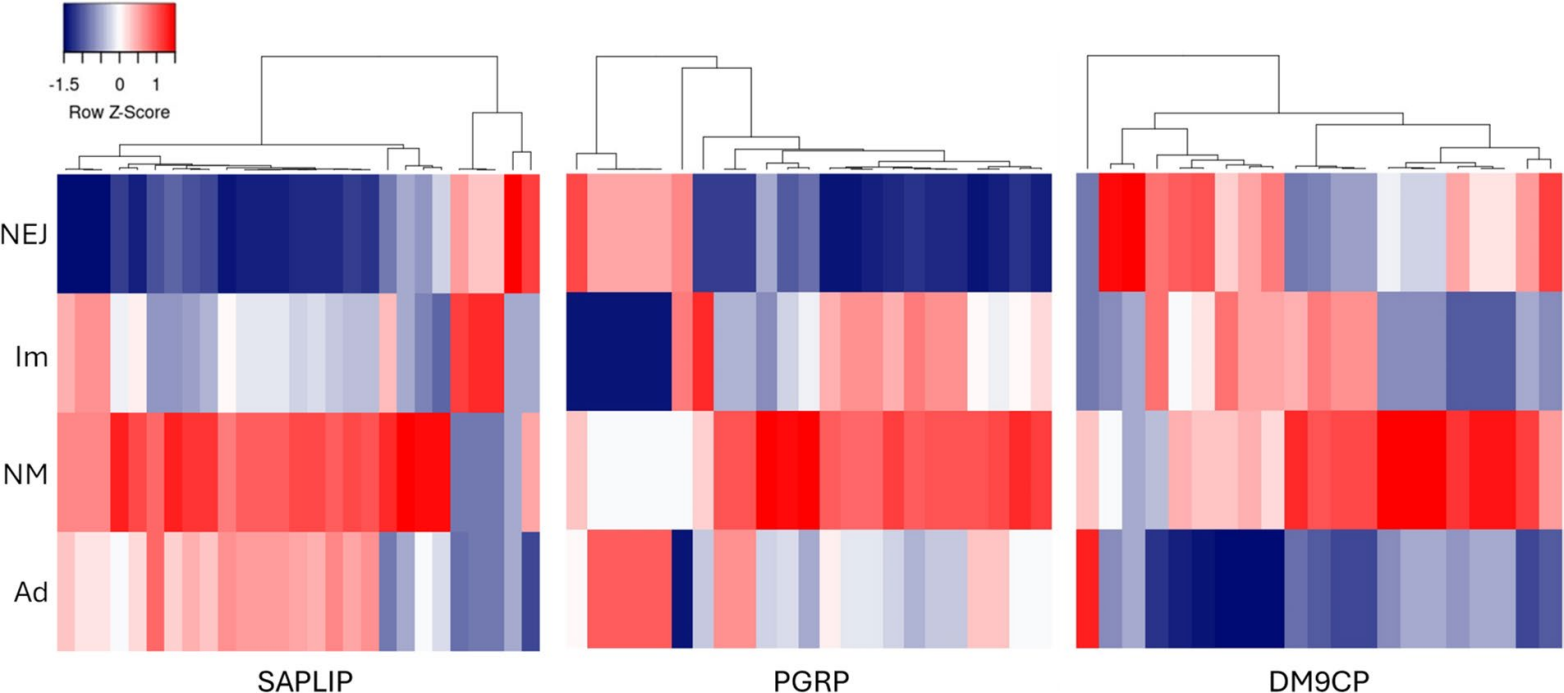
Expression of SAPLIPs, PGRPs and DM9CPs across the intra-mammalian life-cycle stages of *C. daubneyi.* RNA-seq read count data [23] from 4 intra-mammalian life-cycle stages of *C. daubneyi* was mapped to the gene models and expressed as transcripts per million (TPM). Values were represented as a heat map to visualise relative expression levels across a blue to red scale depicting low to high levels of expression, respectively. NEJ, newly-excysted juvenile; Im, immature intestinal flukes; NM, newly migrated flukes; Ad, adult flukes

Changes in gene transcription were followed across the four intra-mammalian developmental stages of *C. daubneyi*, namely the newly excysted juvenile (NEJ) flukes that establish infection in the duodenal submucosa, the immature flukes that feed on host blood/tissue in the intestine, the newly migrated (NM) flukes that have recently arrived in the rumen following their migration from the duodenum and the mature adult flukes that are well-established in the rumen. For this, transcripts per million (TPM) values derived from RNA-seq libraries, in biological triplicate for each stage [23], were mapped to the gene models (Fig. 4). The PGRP, SAPLIP and DM9CP gene families all showed developmental regulation with the highest expression levels generally seen in the infective NEJ stage and in the NM flukes that establish chronic infection within the rumen. Sub-groups of the DM9CPs showed increasing (and overlapping) expression with fluke development, with a clear peak at the NM stage, whilst expression of almost all family members had declined in the adult flukes. The expression of the PGRPs and SAPLIPs were broadly similar with sub-groups showing specific upregulation at the NEJ and immature stages with most family members showing clear upregulation in the NM flukes. In contrast to the DM9CPs, expression of numerous PGRP and SAPLIP genes remained high in the adult flukes.

### Recombinant *C. daubneyi* PGRP binds to bacteria via interaction with peptidoglycans and induces agglutination

**Fig. 5:**
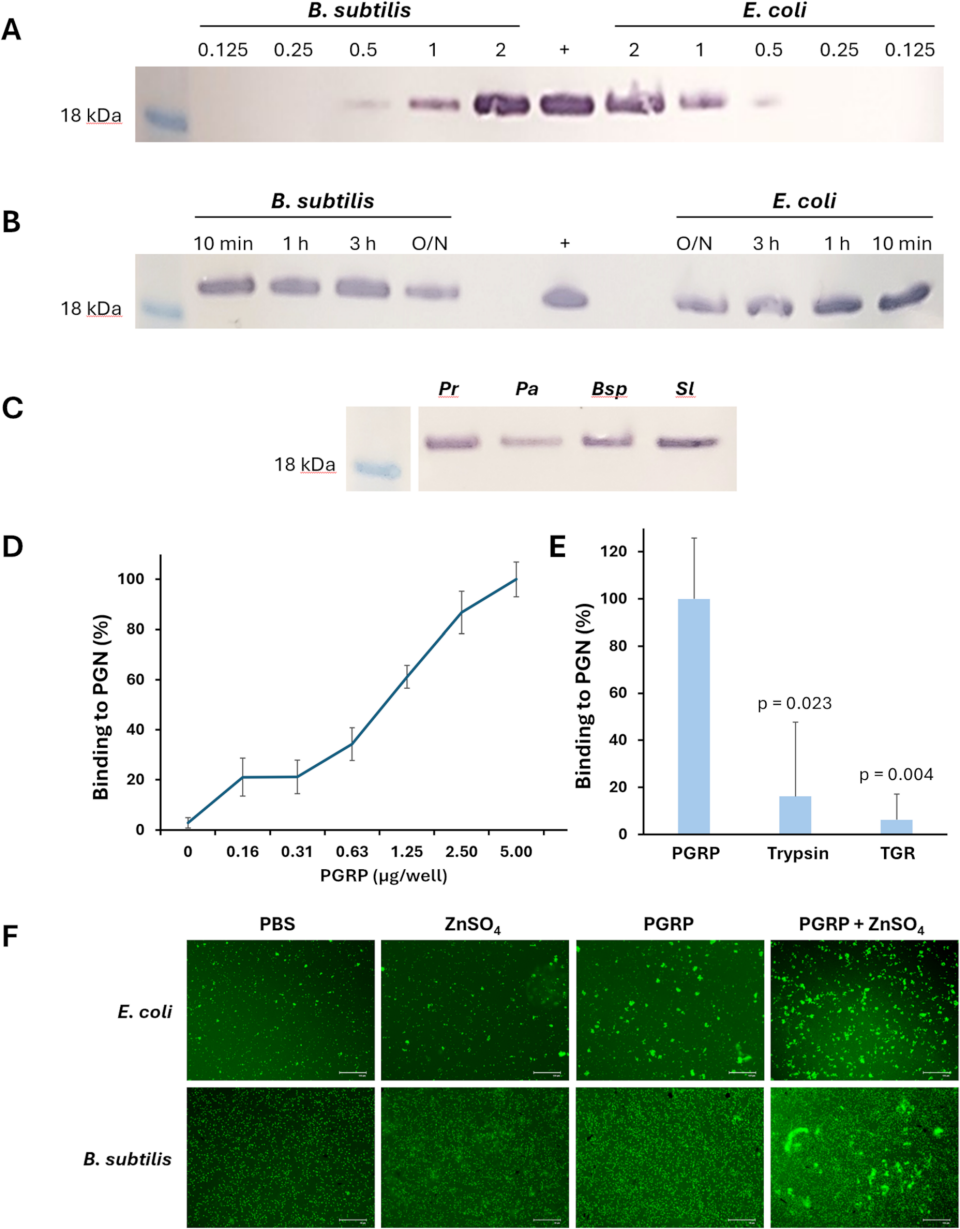
The binding and functional properties of recombinant *C. daubneyi* PGRP. **A ***C. daubneyi* PGRP binds to bacteria in a concentration-dependent manner. Various concentrations (0.125–2 µg) of *C. daubneyi* PGRP were incubated with *E. coli* and *B. subtilis* cells and binding assessed by western blot. One microgram of unbound *C. daubneyi* PGRP was run as a positive control ( +) for the blot. **B** *C. daubneyi* PGRP binds to bacterial cells across multiple incubation periods. PGRP was incubated with *E. coli* and *B. subtilis* cells across various time periods from 10 min to overnight (O/N). Binding was assessed via western blot with 1 µg of unbound *C. daubneyi* PGRP run as a positive control ( +). **C ***C. daubneyi* PGRP binds to multiple species of rumen anaerobes. PGRP was incubated with anaerobic bacterial species commonly found in the rumen and binding assessed by western blot. *Pr*, *P. ruminis*; *Bsp*, *Butyrivibrio spp.*; *Sl*, *S. lutetiensis*; *Pa*, *P. australiense*. Blots shown in panels **A–C** are representative of at least two experiments. **D** The ability of recombinant *C. daubneyi* PGRP to bind peptidoglycan (PGN) was investigated by incubating a range of protein concentrations (0–5 µg/well) in a PGN-coated microtitre plate. Bound proteins were detected by ELISA using rabbit anti-*C. daubneyi* PGRP as a primary antibody. Binding of PGRP to the PGN-immobilised plates was expressed as a percentage of that measured for 5 µg of PGRP (± standard deviation). Data represent means (± standard deviation) of three experiments. **E** Trypsinising the recombinant PGRP significantly reduced the PGN interaction (unpaired *t* test) whilst an un-related recombinant protein (*F. hepatica* thioredoxin glutathione reductase; TGR) showed no specific binding. Data represent means of two experiments. Error bars represent standard deviation. **F** Agglutination of *E. coli* and *B. subtilis* induced by recombinant *C. daubneyi* PGRP. *E. coli* and *B. subtilis* cells were stained with acridine orange and incubated for 45 min at room temperature with recombinant *C. daubneyi* PGRP with or without ZnSO_4_. Bacteria were incubated with PBS or ZnSO_4_ as controls. Agglutination was observed by fluorescence microscopy. Micrographs are representative of two experiments

A full-length *C. daubneyi* PGRP (lacking the predicted N-terminal signal peptide) initially identified within the secretome of adult fluke [23] was expressed in *Escherichia coli* as a ~ 18.7 kDa recombinant protein fused to a C-terminal His_6_-tag for purification. The recombinant PGRP protein was isolated to homogeneity from *E. coli* cell lysates using Ni-chelate affinity chromatography (Additional file 4). The binding of increasing concentrations of recombinant *C. daubneyi* PGRP to model gram negative (*E. coli*) and gram positive (*Bacillus subtilis*) bacterial species was analysed by western blot. PGRP bound to both species in a concentration-dependent manner, with bands of ~ 18 kDa observed with increasing intensity from 0.5 to 2 µg (Fig. 5A).

Further experiments showed that 2 µg of PGRP bound to both species after only 10 min incubation in vitro (Fig. 5B). To determine whether *C. daubneyi* PGRP could interact with biologically relevant species, the binding experiments were also conducted using key anaerobic bacteria found in rumen fluid; namely *Pseudobutyrivibrio ruminis* and *Butyrivibrio spp.* (gram negative) and *Streptococcus lutetiensis* and *Propionibacterium australiense* (gram positive). As seen with the model species, *C. daubneyi* PGRP bound to the surface of all rumen anaerobes tested (Fig. 5C).

Given the binding of *C. daubneyi* PGRP to the surface of bacteria, its specific interaction with peptidoglycan from *Staphylococcus aureus* was determined (Fig. 5D). Recombinant *C. daubneyi* PGRP bound to peptidoglycan in a concentration-dependent manner. Absorbance readings were significantly higher (*p* < 0.05 or *p* < 0.001) when recombinant PGRP was added to peptidoglycan-coated plates compared with uncoated plates, demonstrating the specificity of the interaction. The specific interaction between *C. daubneyi* PGRP and peptidoglycan was additionally supported by a trypsinised PGRP and an un-related recombinant protein, *F. hepatica* thioredoxin glutathione reductase (TGR; expressed/purified in the same manner as PGRP) that displayed 16 and 6% of the peptidoglycan binding shown by PGRP respectively (Fig. 5E). To investigate the functional effect of the binding of *C. daubneyi* PGRP to the bacterial cell surface, *E. coli* and *B. subtilis* were stained with acridine orange and incubated with PGRP, with or without ZnSO_4_, and observed using fluorescence microscopy (Fig. 5F). PGRP induced agglutination of both species in the presence of ZnSO_4_. This effect was also observed for *E. coli* when incubated with PGRP alone, but to a lesser extent. No signs of agglutination were observed when the bacterial cells were incubated with PBS or ZnSO_4_. Although inhibition of growth was observed in the gentamycin control, *C. daubneyi* PGRP showed no direct antimicrobial activity when tested against an ovine ruminal isolate of *S. lutetiensis* during MIC assays.

### The *C. daubneyi* genome suggests an atypical eggshell protein crosslinking chemistry

**Fig. 6:**
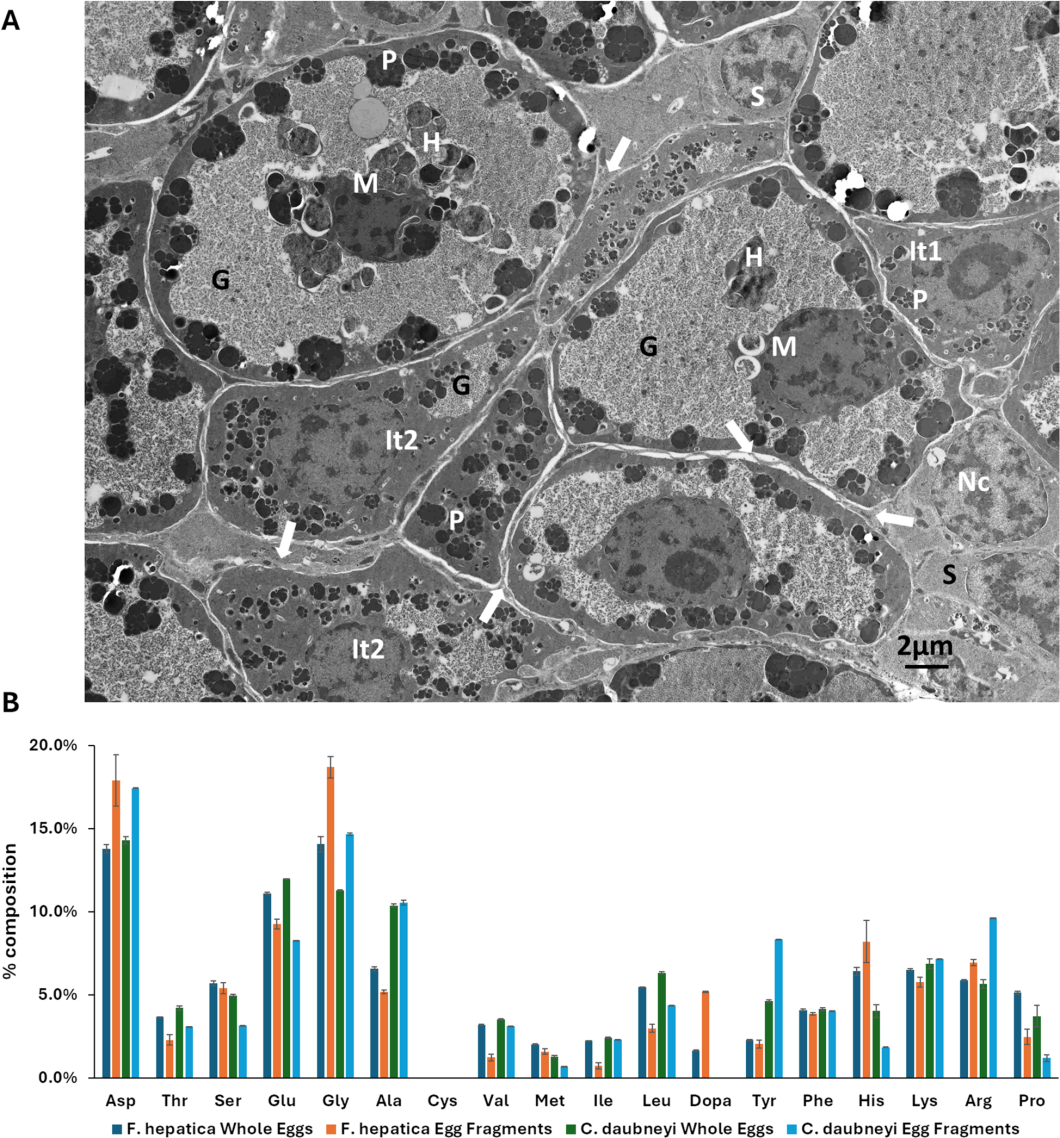
*C. daubneyi* uses atypical eggshell crosslinking chemistry. **A** Transmission electron micrograph of a vitelline follicle of adult *C. daubneyi.* Vitelline cells at various stages of maturation are present, including undifferentiated stem cells (S), early intermediate type cells (It1) in which clusters of shell precursor protein globules (P) are accumulating in the cytoplasm, later intermediate type cells (It2) within which, in addition to shell protein globules, areas of glycogen are apparent (G), and mature vitelline cells (M) with large glycogen reserves, heterophagosomes (H) and peripherally situated clusters of shell protein globules. Cytoplasmic extensions of nurse cells, containing dense mitochondria and lysosomes, envelop the vitelline cells (arrowed), to which they are connected by junctional complexes. Occasional nurse cell bodies are evident, each featuring a nucleus (Nc) with perinuclear cytoplasm. **B** Amino acid composition of *C. daubneyi* and *F. hepatica* whole eggs and eggshell fragments. L-DOPA, which mediates dityrosine crosslinks in trematode eggshells was not detected in *C. daubneyi.* Data are the means ± standard deviation of three experiments

When examining the eggshell-producing vitellaria of *C. daubneyi*, it appears similar to that of other trematodes; specifically, it is arranged in follicles that occupy much of the lateral margins of the fluke. At the ultrastructural level (Fig. 6A), each vitelline follicle is seen to comprise a group of vitelline cells at various stages of development, ranging from undifferentiated stem cells, featuring scant ribosome-rich cytoplasm and euchromatic nuclei, to fully mature vitelline cells. The latter are situated centrally in each follicle and contain numerous peripherally located clusters of dense eggshell protein globules, large cytoplasmic reserves of glycogen, and clusters of heterophagosomes (‘yolk globules’), often located near the nucleus, which is relatively electron dense. Intermediate type vitelline cells exhibit, initially, accumulation of eggshell protein globules in clusters (corresponding to Golgi bodies) within the cytoplasm (It1 cells), followed by marked cell swelling as glycogen reserves amass in the cytoplasm (It2 cells).

Nurse cells, the nuclei of which are often located near the periphery of the follicle, send out numerous dendritic extensions of cytoplasm which surround the developing vitelline cells, making intimate contact with their limiting membrane by means of junctional complexes. Within these cytoplasmic extensions, dense mitochondria and elongated dense lysosomes are present. However, when tissue sections were stained with Mallory’s Trichrome (which stains keratin and collagen), the eggshell protein globule clusters, and the eggshell of whole eggs within the uterus (and those shed by the flukes) of *C. daubneyi* stained a deep red colour whilst those of *F. hepatica* were a golden brown (Additional file 5) suggesting differences in eggshell biochemistry between the two species.

**Fig. 7:**
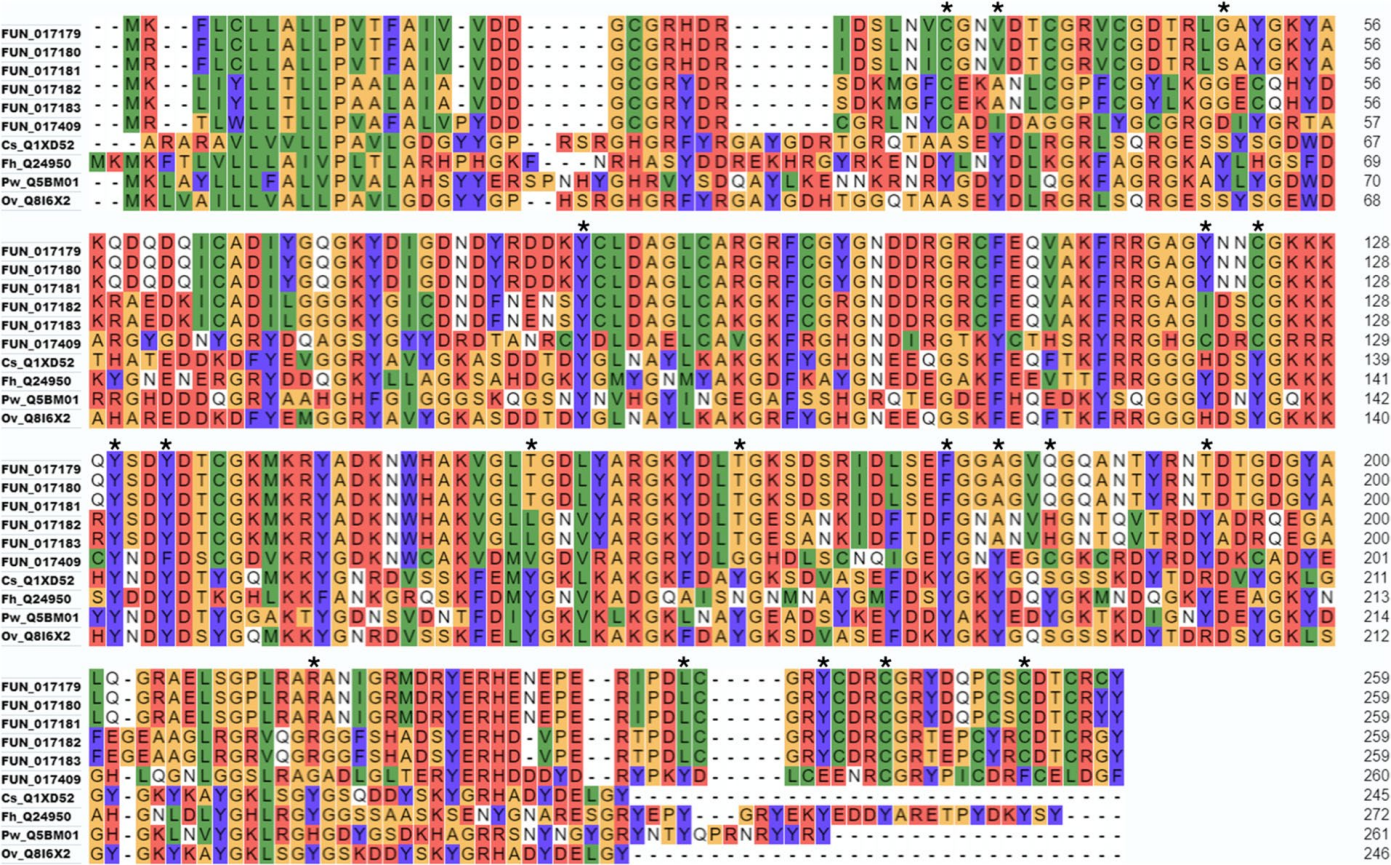
Primary sequence alignment of vitelline proteins from *C. daubneyi* with representative sequences from other trematodes. Fh, *F. hepatica*; Cs, *C. sinensis*; Pw, *P. westermani*; Ov, *O. viverrini*. The position of tyrosine residues converted to L-DOPA in *F. hepatica* vitelline proteins are marked with asterisks. Only 6 out of 19 of these sites were strictly, or partially, conserved in *C. daubneyi*

Trematode eggshells are typically sclerotised by crosslink formation between L-DOPA residues on neighbouring vitelline proteins (reviewed by [44]). There are two schools of thought about DOPA-mediated cross-linking. One posits that DOPA is oxidised to quinone which is attacked by nucleophiles such as lysine and histidine to produce DOPA-Lys/His adducts [45]. This pathway has been confirmed as the mechanism of squid beak hardening [46]. The other, proposes that cross-links arise as *bi*- and *tri*-DOPA-Fe^3+^ complexes and has been best characterised in *Mytilus* byssus [47]. As DOPA formation prior to protein secretion is necessary in both pathways, it is reasonable to expect a protein-directed tyrosyl hydroxylase. BLAST analysis, using *Schistosoma mansoni* tyrosine hydroxylase as query, identified three putative *C. daubneyi* aromatic amino acid hydroxylases; however, backBLAST indicated these were either phenylalanine or tryptophan hydroxylases. In support of this, amino acid analysis found no evidence of L-DOPA in either *C. daubneyi* whole eggs or eggshell fragments whilst *F. hepatica* eggshells contained up to 5.1% L-DOPA residues (Fig. 6B; Additional file 6). Six vitelline protein genes were identified in the *C. daubneyi* genome*.* These typically encoded proteins ~ 29 kDa containing a conserved trematode eggshell synthesis (TES) domain (IPR012615) although one had a predicted mass of 46.4 kDa due to an additional 133 amino acid C-terminal cysteine-, proline- and tyrosine-rich extensin-like region. Three vitelline proteins (including the atypical 46.4 kDa form) were identified within *C. daubneyi* eggshells by mass spectrometry (Additional file 7). Primary sequence analysis showed that the *C. daubneyi* vitelline proteins contained ~ 7% of both tyrosine and cysteine residues and that several tyrosines known to be converted to L-DOPA in *F. hepatica* had been substituted for cysteine (Fig. 7). In contrast, vitelline proteins from other trematodes lack cysteine and contain ~ 12–15% tyrosine residues.

Tyrosinases are often associated with the oxidation of DOPA to DOPAquinone [48]. A single tyrosinase gene was identified in the *C. daubneyi* genome and whilst the functional tyrosinase domain (IPR002227) is present, the enzyme is absent from both of the transcriptome databases generated for adult *C. daubneyi* [22, 23] and that of *P. cervi* [21] and thus is not expressed by the egg-producing stage of paramphistomes. Indeed, the enzyme was not detected during mass spectrometry analysis of *C. daubneyi* eggshell extracts but was found in parallel proteomics analysis of *F. hepatica* eggshells (Additional file 7). Similarly, no staining was observed when adult *C. daubneyi* were incubated with catechol, which visualises tyrosinase as an intense red-brown stain within the vitellaria of *F. hepatica* (Additional file 5; [49]). Taken together, the data suggest that *C. daubneyi* uses different eggshell protein crosslinking chemistry compared to other trematodes.

### The *C. daubneyi* eggshell is resistant to adverse biochemical conditions

Experiments were conducted to determine whether the biochemical differences between *C. daubneyi* and *F. hepatica* eggshells would influence their stability in varied environmental conditions. Initially, whole eggs were collected from both species and exposed to different pH values for 1 or 4 h in vitro and observed by light microscopy. Incubation at pH 4.0 for 4 h led to degradation of the outer layer of the *F. hepatica* eggshell whilst *C. daubneyi* eggs remained intact (Additional file 8). Similar disintegration of the *F. hepatica* eggshell was evident when they were exposed to pH 9.0 for 1 h, and after 4 h incubation very few intact eggs remained. In contrast, *C. daubneyi* eggs remained intact following incubation at pH 9.0, at either time point.

Sodium hypochlorite (NaClO) has been described as a “de-tanning” agent of dityrosine cross-linked trematode eggs and has been shown to cause severe ultrastructural defects to eggs from *S. mansoni* and *F. gigantica* [50, 51]. Here, whole *F. hepatica* and *C. daubneyi* eggs were incubated in increasing concentrations of NaCIO for 1 h and their structural integrity was assessed by light microscopy. No change was observed when either sample was incubated in 0.25% NaCIO solution. However, when incubated in 0.5% NaClO, the *F. hepatica* eggs completely disintegrated whilst the *C. daubneyi* eggs remained intact (Additional file 8). At higher (0.75% and 1.00%) NaClO concentrations, the *F. hepatica* were again completely disintegrated with no visible fragments remaining. In contrast, whilst the *C. daubneyi* eggs were disrupted at these higher concentrations, numerous eggshell fragments were still visible. Collectively, these observations demonstrate that *C. daubneyi* eggs are more resilient than *F. hepatica* eggs when exposed to NaClO and have greater stability across a wider pH range.

### *C. daubneyi* displays specialised gastrodermal cell morphology and a different repertoire of secreted peptidases compared to haematophagous species

**Fig. 8:**
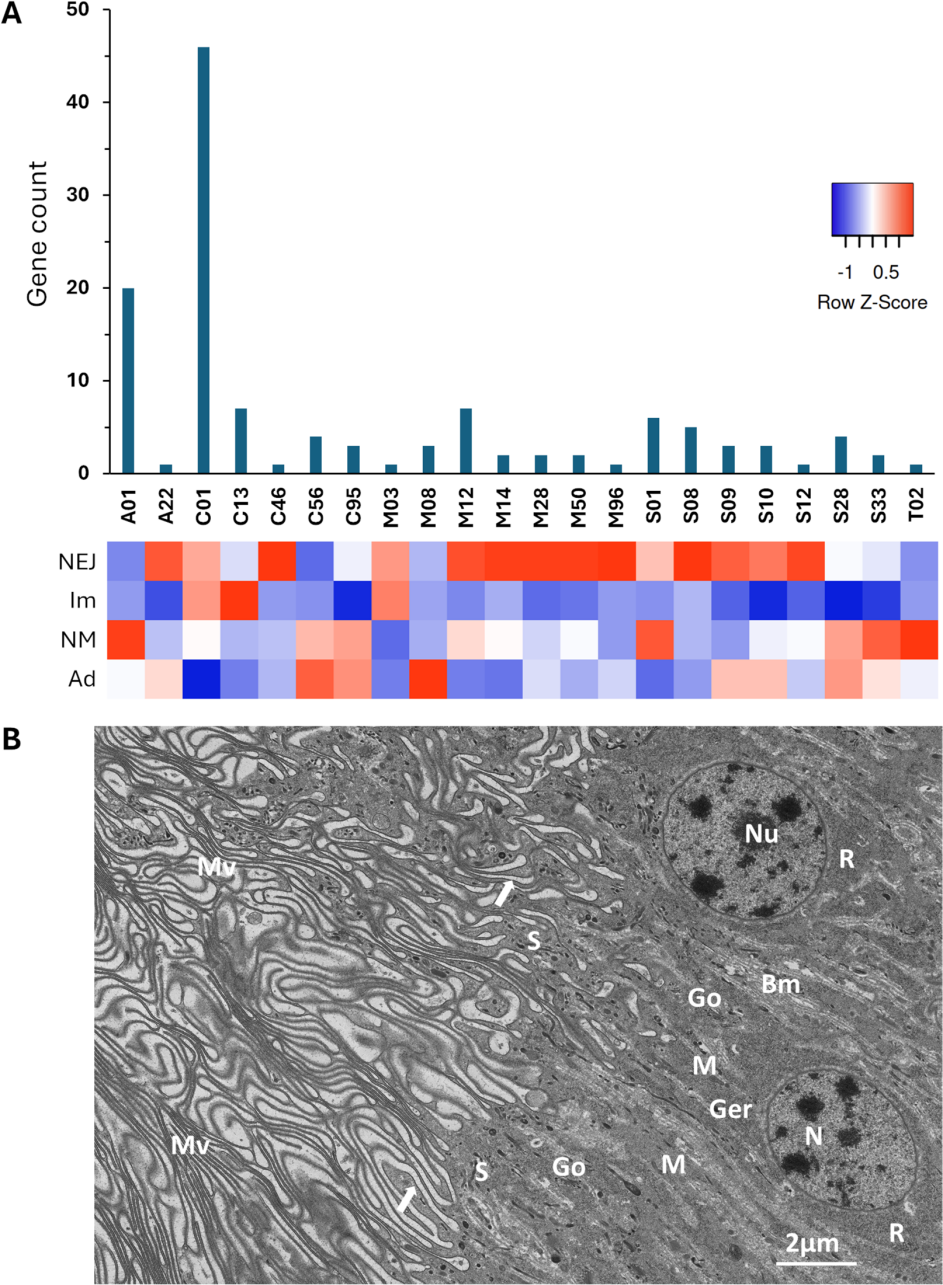
The *C. daubneyi* gut is uniquely adapted for absorption. **A ***C. daubneyi* peptidases bearing an N-terminal signal peptide for secretion via the classical ER/Golgi pathway represent 22 peptidase families but are dominated by the C1A papain-like cysteine peptidases and A1A pepsin-like aspartic peptidases. Total TPM values [23] for each peptidase family were represented as a heat map (with a blue to red scale depicting low to high levels of expression, respectively) to visualise relative expression across the intra-mammalian life-cycle stages of *C. daubneyi.* NEJ, newly excysted juvenile; Im, immature intestinal flukes; NM, newly-migrated flukes; Ad, adult flukes. **B** Transmission electron micrograph of secretory cells comprising the gastrodermis in adult *C. daubneyi*. The elongated cells feature centrally situated, spheroidal euchromatic nuclei (N), each with a large nucleolus (Nu). The lateral plasma membranes are difficult to distinguish amongst elaborate infolds of the basal membranes (Bm), which reach to the apical cytoplasm. Granular endoplasmic reticulum (Ger) together with free ribosomes (R) predominate in the basal and perinuclear cytoplasm, whilst Golgi bodies (Go), each with dense secretory vesicles associated with the maturing face, occur frequently in the apical cytoplasm. These secretory vesicles form larger spherical to elongate secretory bodies towards the cell apex (S). Small mitochondria (M) of moderate electron density are numerous but inconspicuous. The apex of each cell is highly elaborated by numerous, long, densely packed and sometimes branching microvilli (Mv). Adjacent microvilli are sometimes connected by fine fibrous bridges (arrowed) emanating from the glycocalyx

Histological examination of adult *C. daubneyi*, that were fixed immediately after removal from the host rumen, revealed the presence of ciliates within the oral cavity/foregut. However, recognisable structure is rapidly lost as these move deeper within the gut caeca (Additional file 9), consistent with extracellular digestion. A total of 423 peptidases were identified in the *C. daubneyi* genome, of which 126 encoded an N-terminal signal peptide for secretion via the classical ER/Golgi pathway. These were dominated by the C1A papain-like cysteine peptidases (47 sequences) which comprised 43 cathepsins B (representing 38 genes and 5 isoforms), four putative cathepsin L/F genes and a single dipeptidylpeptidase (cathepsin C) gene. Other abundant groups of secreted peptidases included the A1A pepsin-like aspartic peptidases (15 genes and 5 isoforms) and the C13 legumain-like asparaginyl endopeptidases (5 genes and 2 isoforms) (Fig. 8A).

The cells lining the *C. daubneyi* gut exhibit ultrastructural features associated with secretory and absorptive activity, whilst the apex of each cell bears numerous microvillae/lamellae, each approximately 50 nm thick, which are densely packed and extend far into the gut lumen (Fig. 8B). The limiting membranes of these bear a dense glycocalyx from which fibrous strands emanate, often connecting adjoining lamellae.

The apical cytoplasm of the gastrodermal cells features Golgi bodies, each with 4–5 elongated sacs per Golgi stack, and small dense secretory vesicles are associated with the maturing face. These dense vesicles apparently join to give rise to larger spherical to elongated secretory bodies below the apical membrane. These discharge their contents at the base of the microvilli/lamellae, as evidenced by the appearance of temporary depressions at the cell surface. Predominant in the cytoplasm of the basal and perinuclear regions in each gastrodermal cell are free ribosomes and cisternae of granular endoplasmic reticulum. Mitochondria, though numerous, are small, inconspicuous and of moderate electron density. The basal plasma membranes of the gastrodermal cells are extensively infolded, forming lamellae that extend to the perinuclear and apical cytoplasm.

The extensive lamellae/microvillae that extend from the apical surface of the gastrodermal cells of *C. daubneyi* would provide a huge surface area for absorption of molecules, such as volatile fatty acids (VFAs), that are abundant in rumen fluid. In mammalian gastrointestinal epithelial cells, these are imported via transport proteins expressed on the plasma membrane that include the H^+^-coupled MCT1 (SLC16A1) and MCT4 (SLC16A3) transporters and the Na^+^-coupled SMCT1 (SLC5A8) and SMCT2 (SLC5A12) transporters [52]. Whilst BLAST searches using human VFA transporters as queries identified 12 and 6 genes in *C. daubneyi* encoding putative H^+^-coupled (IPR050327; proton-linked MCT) and Na^+^-coupled (IPR051163; sodium:solute symporter) transporters respectively, similar genes were also identified in *F. hepatica*, an obligate blood-feeder.

## Discussion

Clinical paramphistomosis, associated with large numbers of immature *C. daubneyi* in the duodenal submucosa, can cause significant damage to host tissue [5] and may result in fatal disease outbreaks in both cattle and sheep [6, 8, 53]. In contrast, chronic infection caused by adult flukes residing within the rumen is seemingly well-tolerated in terms of animal health and productivity [9, 16, 54] even with very heavy parasite burdens (e.g. > 1000 flukes per cow; [16]) which are frequently observed. The rumen niche is biochemically unique compared with the sites typically occupied by other trematode species (e.g. bile duct, blood vessels, lung tissue). Moreover, rumen fluid is particularly microbiome-rich containing a vast array of bacteria, fungi, ciliates and viruses [17]. However, it remains unclear how *C. daubneyi* interacts with, and modulates, these microorganisms with which it shares the rumen epithelial microenvironment. To begin to unravel such interactions, and other poorly understood areas of rumen fluke biology, we present a 1.76-Gb draft genome for *C. daubneyi*, the first to be described for any paramphistome species. The genomic data, integrated with microscopical, biochemical and proteomics investigations reveal a number of novel molecular and morphological adaptations for microbial recognition/defence, feeding and reproduction that enable *C. daubneyi* to thrive in such a hostile host environment.

The BUSCO scores of the final *C. daubneyi* 1.76 Gb genome demonstrate that our assembly is amongst the largest and highest quality reported for any trematode species to date. This is likely due to the use of the PacBio sequencing platform for both the genome sequencing and supporting RNA evidence [23] which generates much longer, better quality reads than other sequencing technologies. The availability of the genome resources allowed the first phylogenomic-level analysis to include a paramphistome to be performed using 180 shared single-copy orthologues from *C. daubneyi* and 17 other flatworms. In the resulting tree, the phylogenetic relationships of free-living flatworms, monogeneans, cestodes and trematodes were in agreement with a previous genome-wide phylogeny of 25 platyhelminth species [55] with the addition of *C. daubneyi* (representing the paramphistomidae) which segregated from other sub-order Plagiorchiida trematodes, such as *F. hepatica*, after the split from the schistosome lineage. Whilst *C. daubneyi* showed similar coverage of KEGG metabolic pathways as phylogenetically related species, a number of gene families, with putative roles in bacterial recognition/defence, have undergone significant expansion. Given, the microbial milieu in which *C. daubneyi* reside, an expansion of gene families with such roles is perhaps not surprising. Despite this, little is known with regard to the impact of rumen fluke on the host microbiome with a solitary investigation into rumen fluke EVs [19] providing our only insight to date.

Peptidoglycan recognition proteins (PGRPs) are a highly conserved family of pattern recognition receptors (PRR) first described in the silkworm, *Bombyx mori* [56]. They exist in short (secretory) and long (transmembrane/secretory) forms and are considered key components of the innate immune system as they recognise pathogen-associated molecular patterns (PAMPs), such as peptidoglycan (PGN), on bacterial cell walls [39]. PGRPs are classified according to their biochemical activity. Catalytic PGRPs possess amidase activity and can cleave the amide bond between *N*-acetylmuramic acid and L-alanine of PGN [57]. This can lead to loss of cell wall integrity and may be responsible for the direct bactericidal effects of some insect and mammalian PGRPs [39, 58]. In contrast, non-catalytic PGRPs (typically seen in invertebrates) lack enzymatic activity but, after binding to PGN, activate the Imd, Toll and prophenoloxidase (PPO) pathways, the three major immune effector mechanisms in insects [58–60]. The *C. daubneyi* genome contains 18 PGRP genes with an additional 5 isoforms, which in comparison to other parasitic flatworm species (with 0–4 PGRP genes), represents a major expansion of this family. We previously identified two short form PGRPs in the secretome of adult *C. daubneyi* using mass spectrometry [23], the most abundant of which was produced as an ~ 18.7 kDa recombinant protein in *E. coli*. The recombinant PGRP bound rapidly to the surface of gram-positive and gram-negative aerobic bacteria, and to several obligate anaerobes typically found in the rumen microbiome, via specific interaction with purified cell wall peptidoglycans. Moreover, the recombinant PGRP had a moderate agglutinating effect on bacteria which was enhanced in the presence of Zn^2+^ ions, a co-factor of catalytic PGRPs [61]. Conceivably, this action could confer protection against colonisation of the fluke tissues by pathogenic bacteria but may also facilitate ingestion of bacterial cells, in clusters, as a source of nutrition (see below). Although the recombinant PGRP showed no direct antimicrobial activity against *S. lutetiensis* during MIC assays, it is possible that *C. daubneyi* PGRPs work synergistically with other secreted molecules to achieve an antimicrobial effect.

Comparative genome analysis also revealed that the *C. daubneyi* saposin-like protein (SAPLIP) family has undergone significant, and specific, expansion (26 genes and 4 additional isoforms) compared to other flatworm species (with 0–12 genes). SAPLIPs are members of the amoebapore superfamily of lipid-binding molecules that interact with biological membranes in one of three ways: binding, perturbation without permeabilization, or permeabilization [62]. In blood-feeding trematodes, such as *F. hepatica* and *C. sinensis*, SAPLIPs have been shown to induce lysis of erythrocytes and release of haemoglobin which can be degraded by secreted peptidases [63–65]. However, many SAPLIPs are important effectors of the innate immune system, with roles in pathogen recognition, membrane permeabilization and cellular lysis [62]. Indeed, many of the *C. daubneyi* SAPLIP genes showed particular sequence identity with NK lysins which are SAPLIP family members, typically characterised from vertebrates, that show antimicrobial activity against a broad range of pathogens including fungi, protozoa and bacteria (reviewed by [66]). Since adult paramphistomes do not appear to be haematophagous (see below), it is likely that the large SAPLIP family of *C. daubneyi* has evolved as a means of defence against the vast numbers, and great diversity, of microorganisms present within the rumen environment. As SAPLIPs are also present in *C. daubneyi* soluble secretions [23] and EVs (Morphew & Robinson, unpublished data), it is conceivable that they work in concert with the PGRPs to disrupt the bacterial cell wall. In this scenario, the putative amidase activity of the PGRPs would first degrade the outer peptidoglycan layer and thus allow the SAPLIPs to access, and lyse, the underlying phospholipid membrane. Continued investigation of the function of *C. daubneyi* secretory molecules will help answer one of four key questions posed recently by Rooney et al. [67] in understanding the mechanisms of helminth-microbiome interactions.

A family of DM9 domain-containing proteins (DM9CPs) have also undergone expansion in *C. daubneyi* (20 genes and 2 additional isoforms) compared with other flatworm species (0–9 genes). The DM9CPs are mainly found in arthropods and platyhelminths [68], and typically possess two or more DM9 domains which contain a 60–75 amino acid motif first identified in the fruit fly, *Drosophila melanogaster* [42, 69]. Although not well characterised, recent studies have shown that oyster and crab-derived DM9CPs have roles in innate immunity and can induce agglutination of bacterial and fungal cells and enhance the encapsulation activity of haemocytes. DM9CPs can also bind to a variety of PAMPs such as mannose, lipopolysaccharide and peptidoglycan and are thought to act as multipotent PRRs [70–72]. DM9CPs were identified within the protein cargo of EVs secreted by *C. daubneyi* [19], *S. mansoni* [73] and *F. hepatica* where they were specifically found on the EV surface [74]. Although the function of the *C. daubneyi* DM9CPs has yet to be determined, an orthologue from *F. gigantica* displayed agglutination activity against gram-negative and gram-positive bacteria [75]. As for the PGRPs and SAPLIPs, it is likely that the unique microbial-associated selective pressures exerted on *C. daubneyi* have driven the expansion of a DM9CP family with sensory/protective functions. Gene expression analysis suggests that the PGRPs, SAPLIPs and, to a lesser extent, the DM9CPs, cluster according to those upregulated in the NEJ and immature fluke stages (which inhabit the duodenum) and those upregulated in the newly migrated and adult flukes (which inhabit the rumen). Since the composition of the host microbiome varies greatly between these locations [76], it appears that the gene expansions have created large families of secretory molecules conferring (potentially synergistic) recognition of/defence against a wide range of bacteria concomitant with fluke development and migration.

Adult *C. daubneyi* attach to the rumen epithelium via a muscular posterior sucker. This arrangement ensures that the oral sucker, and thus the opening to the blind-ended gut, is free to move within the rumen fluid [9]. The general absence of red blood cells in the gut of paramphistomes [77–79] and the lack of significant pathology within the rumen epithelium suggests that adult rumen flukes are not obligate blood-feeders [53, 80]. The detection of cellular debris, bacteria and ciliates, derived from rumen fluid, within the pharynx and oesophagus of *C. daubneyi* suggests that they feed by direct digestion of the rumen microbial community [80]. In support of this, our microscopical observations of rumen fluke tissue sections suggest that adult *C. daubneyi* are capable of extracellular digestion of rumen microorganisms. Indeed, UK-sourced paramphistomes, identified as *Paramphistomum hiberniae* in the 1950s (which may have been misidentified *C. daubneyi* specimens; [20]), could be maintained in rumen fluid alone for up to 9 days in vitro [81]. However, *C. daubneyi* had no effect on ciliate numbers when co-cultured in vitro [22].

Ultrastructural examination of the gastrodermal cells that line the *C. daubneyi* gut revealed features associated with secretory and absorptive activity. Transcriptome and secretome data demonstrate that whilst *C. daubneyi* adults secrete a very different repertoire of digestive peptidases compared to blood-feeding adult *F. hepatica* (i.e. cathepsins B and aspartic peptidases rather than cathepsins L1/2/5; [23, 82], they are not particularly abundant. This is in contrast to liver fluke where cathepsins L comprise around 80% of the total protein secreted by the adult stage [82, 83] and may be linked to the abundance of free amino acids in rumen fluid for direct uptake [84]. Collectively, these observations suggest that extracellular digestion may not be the sole source of nutrient acquisition by *C. daubneyi* residing within the rumen. The dense lamellae/microvillae that extend from the apical surface of *C. daubneyi* gastrodermal cells would provide a huge surface area for absorption of molecules from the rumen fluid. Indeed, the ruminant host primarily utilises volatile fatty acids (VFAs) such as acetate, propionate and butyrate that are produced during microbial fermentation in the rumen as a source of energy [85]. In mammalian gastrointestinal epithelial cells, these are typically imported via transport proteins expressed on the plasma membrane that include the H^+^-coupled MCT1 (SLC16A1) and MCT4 (SLC16A3) transporters and the Na^+^-coupled SMCT1 (SLC5A8) and SMCT2 (SLC5A12) transporters [52]. Whilst putative homologues of these VFA transporters were identified within the *C. daubneyi* genome, similar genes were also observed in *F. hepatica* demonstrating that they are not exclusive to rumen-dwelling paramphistomes. However, the gastrodermal cell lamellae/microvillae of *C. daubneyi* are around three times longer than those of *F. hepatica* [77] which would yield a much larger surface area for expression of VFA transporters and VFA uptake from rumen fluid.

Despite the apparent differences in the feeding mechanism used by *C. daubneyi* compared with other trematodes, all share the same nutritional requirement—to support the production of vast numbers of eggs; e.g. *F. hepatica* produces an average of 25,000 eggs per fluke, per day [86]. Trematode eggshells are comprised of vitelline proteins, released from protein globule clusters in mature vitelline cells, that coalesce around the ovum [44]. The vitelline proteins form a tough protective layer around the developing embryo in a process that has been known as quinone tanning, during which tyrosine residues present in the vitelline proteins are initially modified by the addition of a hydroxyl group to form L-DOPA (3,4-dihydroxyphenyl-l-alanine), in a step catalysed by tyrosine hydroxylase. The L-DOPA residues are presumed to be oxidised by tyrosinase/phenol oxidases to form DOPA-Lys and/or DOPA-His crosslinks between neighbouring vitelline proteins which serves to harden and stabilise the eggshell [46, 87, 88]. This scenario, however, is being questioned by the finding that in *Mytilus* byssus most of the DOPA is cross-linked by bis- and tris-coordination of Fe^3+^ [47]. Given that trematodes like *Fasciola *spp. and *Schistosoma *spp. consume blood with iron-rich haemoglobin, they may rely on it as well [89]. At the ultrastructural level, *C. daubneyi* vitelline follicles appear similar to that of other trematodes and contain vitelline cells at progressive stages of development that culminate in mature cells bearing numerous eggshell protein globule clusters within their peripheral cytoplasm. However, our data suggest that *C. daubneyi* uses atypical eggshell protein crosslinking chemistry. Firstly, whilst amino acid analysis revealed up to 5.1% L-DOPA residues in *F. hepatica* eggshell proteins, L-DOPA was not detected in *C. daubneyi* eggshells, likely due to the lack of a tyrosine hydroxylase gene which catalyses the conversion of tyrosine to L-DOPA. Indeed, alignment of six conceptually translated *C. daubneyi* vitelline protein genes showed that several tyrosine residues, known to be converted to L-DOPA in *F. hepatica* [90], were substituted for cysteine which is not found in the vitelline proteins of other trematodes. Secondly, although a single gene encoding the putative eggshell crosslinking enzyme, tyrosinase, was identified in the *C. daubneyi* genome, RNA-seq data showed that it is not expressed by egg-producing adult flukes. In addition, the enzyme was not detected during MS/MS analysis of rumen fluke eggshell extracts. Previous histochemical studies of other paramphistome species have suggested that their eggshells are keratinised and are stabilised by disulphide (Cys-Cys) bonds rather than via dityrosine crosslinks [91]. Cysteine-rich eggshell membrane proteins are common in avian and reptilian eggshells and are disulfide crosslinked in the mature membranes [92]. Despite their occurrence in *C. daubneyi* vitelline protein primary sequences (~ 7%), cysteines were not detected in their eggshells by direct biochemical analysis. Thiol cysteines do not typically survive the acid hydrolysis extraction procedure used during amino acid analysis, but disulphide cysteines do, so the absence of cysteines suggests that disulphide crosslinks may not occur in *C. daubneyi* eggshells.

Mass spectrometry analysis of *C. daubneyi* eggshells led to the identification of an uncharacterised 113 kDa secretory protein with an internal region showing ~ 37% sequence identity with a proline-rich extensin-like protein. Moreover, one of the rumen fluke vitelline proteins (also confirmed within the eggshell by mass spectrometry) also possessed a C-terminal extension with ~ 30% sequence identity with an extensin-like protein. Extensins are important structural components of plant cell walls which form intramolecular isodityrosine crosslinks and intermolecular di-isodityrosine crosslinks, catalysed by extensin peroxidases, at Tyr-Xaa-Tyr motifs [93, 94]. The vitelline protein C-terminal region contains 4 Tyr-Xaa-Tyr motifs whilst a thioredoxin domain-containing protein 17, with putative peroxidase activity [95] was detected in eggshell protein extracts by mass spectrometry. Although requiring experimental validation, peroxidase-mediated di-isodityrosine crosslinks between adjacent vitelline proteins may stabilise the eggshell and contribute to the enhanced stability of *C. daubneyi* eggs. Furthermore, the large number of ferritins identified during MS/MS analysis of *C. daubneyi* eggshell extracts suggests that catechol-iron coordination could play a role in eggshell hardening as suggested for *S. mansoni* [96]. Due to the heteropolymeric nature of trematode eggshells, it is perhaps likely that multiple intermolecular bonds participate in this process.

## Conclusions

Our analysis of the *C. daubneyi* draft genome has allowed us to investigate key aspects of paramphistome biology like never before. One of the defining features of *C. daubneyi* is its final niche within its mammalian host—the rumen. The rumen maintains a highly complex ecosystem of microorganisms that break down plant-based feed thus increasing nutrient availability to the host [17]. In particular, enteric fermentation by methanogenic archaea within the rumen is the leading cause of methane (CH_4_) emissions from ruminant livestock which contributes substantially to global greenhouse gas (GHG) emissions (reviewed by [24]). Our genome-led discovery of a family of *C. daubneyi*-derived PGRPs that can bind to the bacterial cell surface and induce agglutination represents a novel route for helminth-microbe interaction. Further characterisation of the putative antibacterial effects of PGRPs, and the similarly expanded SAPLIP family, will lead to a greater understanding of how paramphistomes modulate microbial communities within the rumen and perhaps influence enteric CH_4_ emissions from livestock [97]. This is not without precedent as the considerable increases in CH_4_ emissions yield seen with *Haemonchus contortus* and *Trichostrongylus colubriformis* infections in sheep were also associated with changes in rumen bacterial diversity—characterised by suppression of species that regulate ruminal homeostasis and expansion of methanogenic archaea [98].

In addition to direct antimicrobial effects, molecules secreted by *C. daubneyi* may also influence how host cells sense, and respond to, bacterial PAMPs. Detection of PAMPs by PRRs, such as toll-like receptors (TLRs) expressed by host epithelial cells and/or innate immune cells leads to inflammatory responses, the resolution of which results in tolerance to commensal microbes or clearance of pathogens [89]. We have found that *C. daubneyi* secretes an expanded repertoire of putative PRRs (PGRPs and DM9CPs) with confirmed/predicted ability to bind bacterial ligands such as peptidoglycan and lipopolysaccharide (LPS). In addition, our previous mass spectrometry analysis revealed that helminth defence molecule (HDM) and fatty-acid binding proteins (FABPs) were amongst the most abundant proteins in the rumen fluke secretome [22, 23]. Strikingly, orthologues of both molecules block TLR4 signalling and protected mice against LPS-induced inflammation by significantly reducing levels of TNFα and IL-1β [99–102]. Thus, it is conceivable that the array of PRRs secreted by *C. daubneyi* will regulate inflammatory responses within the rumen, resulting in altered microbial diversity. Although adult *C. daubneyi* are not overtly pathogenic, such dysregulation of the rumen microbiome can influence ruminal fermentation processes, which are critical for the animal’s nutrient absorption and energy production, thereby reducing feed utilisation efficiency. This disruption may have indirect, yet significant, implications for animal health and productivity.

By reporting the draft genome for *C. daubneyi*, we have created a platform for comparative genome-level studies with other rumen-dwelling paramphistomes such as *P. cervi*, *Gastrothylax crumenifer*, *Cotylophoron cotylophorum* and *Fischoederius elongates* which are considered production-limiting infections of livestock in tropical/sub-tropical regions [11, 12, 103]. The continued generation, and exploitation, of multi-omics resources for paramphistomes should lead to the discovery of novel targets for molecular diagnostic tests for pre-patent infection (associated with the most damaging pathology). This would be a major advance on current microscopical methods and would help avoid economic losses associated with paramphistomosis, especially in low/middle-income countries.

## Methods

### Parasite material and DNA sequencing

Adult *C. daubneyi* were collected from the rumen of a naturally infected cow in a local abattoir (Dungannon, Northern Ireland). Flukes were washed several times in warm (39 °C) sterile PBS, snap-frozen in liquid nitrogen and stored at − 80 °C until use. High molecular weight genomic DNA was isolated using the Qiagen Genomic Tip 100/G kit and quantified/quality-controlled using a Qubit fluorometer (ThermoFisher) and agarose gel electrophoresis. Hi-Fi libraries were prepared using the SMRTbell Express Template Prep Kit 2.0 (PacBio). Five micrograms genomic DNA (from a single fluke) was sheared to an appropriate size (15–20 kb) with the Megaruptor 3 (Digenode). This material was made free of single-stranded ends, damage repaired and end tailed. This was ligated to an overhang adapter to create the SMRT bell library. Non-circular molecules were removed in an enzyme clean up step. The DNA was size selected using a 0.75% agarose cassette and s1 marker using the Sage Blue Pippin system and sequenced using Sequel Polymerase 2.2 on the PacBio Sequel 2e platform using 30-h movies and adaptative loading.

### Genome assembly

PacBio HiFi reads were generated by SMRTlink 10 [104] and subsequently assembled by HiFiASM [105] using default settings. The genome assembly was screened for contamination using Blobtools2 [106] using default settings. The completeness of the assembly was assessed by BUSCO [107] using Eukaryota Odb10 [108]. Prior to gene prediction, repeat models were generated using Red [109], which were subsequently used as BLASTX queries against the UniProt/Swiss-Prot database of curated proteins. In order to improve the sensitivity of the gene prediction process, sequences that significantly matched with any protein were removed from the repeat models using ProtExcluder [110]. Finally, filtered repeat models were applied to the genome, using RepeatMasker [111].

### Gene prediction and annotation

Genes were predicted using the Funannotate pipeline which uses both protein and RNA evidence to train a suite of ab initio predictor tools: AUGUSTUS [112], Snap [113], GeneMark [114], GlimmerHMM [115] and PASA [116]. Here, the UniProt/Swiss-Prot curated protein dataset was used as protein evidence, and our previous long-read Isoseq and short-read RNA-seq data for *C. daubneyi* [23] was provided as transcript evidence. The protein data was aligned to the genome using Diamond BLASTX [117] to identify strong matches to coding sequences. RNA-seq data were aligned to the genome using the splice-aware short-read alignment tool, HISAT2 [118], with a maximum intron size of 300 kb. Aligned data were used to generate a reference-guided transcriptome assembly with Trinity [119] that was post-processed with Stringtie [120] and PASA. The above steps were carried out using the Funannotate train1 module, and the resulting filtered, high-quality set of transcript and protein alignments was used in the subsequent training of the gene predictors. As an additional option, the module was instructed to carry out optimisation of AUGUSTUS gene models. Predictions from each tool were collated and filtered by the Funannotate evidence modeller (EVM) to yield a final set of genes. Gene models were refined (splitting/fusing of gene models, and prediction of putative transcript isoforms) and updated to add predicted UTR sequences, based on RNA evidence. Genes were annotated using the Funannotate annotate module, which uses the Eggnog-mapper [121], UniProt MEROPS and PFAM to generate functional predictions where possible. Finally, to correlate gene expression with parasite development, RNA-seq read count data from 4 intra-mammalian life-cycle stages of *C. daubneyi* (NEJs, immature flukes, newly migrated flukes and adults; identified by gross morphology, histological examination and location within the mammalian host [23]) was mapped to the gene models and expressed as transcripts per million (TPM) as previously described [23].

### Phylogenetic analysis

Phylogenetic tree generation was performed using a range of platyhelminth genomes and *Homo sapiens* as an outgroup. Single-copy orthologue groups (Additional file 10) were identified between all species using Orthofinder (v2.5.4) [122]. Protein sequences within single-copy orthogroups were aligned using maaft (v7.487) [123], followed by alignment trimming with gblocks (0.91b) [124]. All alignments were concatenated and provided to modeltest-ng-static (v0.1.7) [125] for amino-acid substitution model testing. Finally, the alignment and selected model were provided to RaxML-ng (v0.6.0) [126] for tree inference. RaxML-ng was performed using 10 randomised parsimony starting trees and 1000 bootstrap replicates.

### Comparative genomics of platyhelminth species

A number of the best-quality platyhelminth genomes were selected for comparison with the *C. daubneyi* genome assembly using a similar approach to Coghlan et al. [55]. Species, representing the major clades, were selected based on BUSCO (v5.2.2) [107] proteome score (> 80% completeness, eukaryota_odb10). Longest iso-forms were chosen as a representative for each gene, which prevents false positives when studying gene expansion/contraction. Orthogroups were identified across all species using Orthofinder, with newick tree generation again described as above. Changes in gene family numbers were assessed two ways: (1) using CAFE (v5) [127], and (2) GLM analysis using Maaslin2 (v1.6.0) [128] using the “ZINB” option, a zero inflation model to take into account the high number of zeros for some species in orthogroups. A final set of expanded or contracted gene families were taken as those that were significant in both analyses. *C. daubenyi g*enes were annotated with gene ontology (GO) terms using eggnog (v5.0) [129].

### Identification, and phylogenetic analysis, of *C. daubneyi* gene families

*C. daubneyi* gene families and specific homologues of interest were identified using BlastP (1.0 × 10^−6^ e-value cut-off) run locally within BioEdit [130] using query sequences from other trematodes (e.g. *F. hepatica, Opisthorchis viverrini, Clonorchis sinensis* and *Schistosoma spp*.). TPM values for each gene across 4 intra-mammalian lifecycle stages, assigned using previous RNA-seq data [23], were used to produce heatmaps of gene expression via the heatmapper.ca expression tool [131]. Neighbour-Joining phylogenetic trees were constructed for selected protein sequences using MEGAX [132]. The bootstrap consensus tree inferred from 1000 replicates was taken to represent the evolutionary history of the taxa analysed [133]. Branches corresponding to partitions reproduced in less than 50% bootstrap replicates were collapsed. The percentage of replicate trees in which the associated taxa clustered together in the bootstrap test (1000 replicates) were shown next to the branches. The evolutionary distances were computed using the Poisson correction method [134] and are in the units of the number of amino acid substitutions per site.

### Expression and purification of recombinant *C. daubneyi* PGRP

Recombinant *C. daubneyi* PGRP was produced in *Escherichia coli* by Genscript (NJ, USA). Briefly the protein coding sequence of transcript Cdaub_10767, the most abundant PGRP found in the *C. daubneyi* secretome [23], was codon-optimised for *E. coli*, chemically synthesised, and subcloned into the pET30a expression vector. *E. coli* BL21 Star (DE3) cells were transformed with the construct and bacterial cultures (1 L) were grown in LB media supplemented with kanamycin (100 mg/ml) at 37 °C until OD_600_ 0.6–0.8 and expression of the recombinant PGRP was induced by the addition of 0.5 mM IPTG for 16 h at 15 °C. Soluble His-tagged recombinant PGRP was purified from cell lysates by Ni–NTA affinity chromatography, buffer exchanged into 50 mM Tris–HCl, 500 mM NaCl, 10% v/v glycerol (pH 8.0) and stored in aliquots at − 20 °C until use.

### Binding of recombinant PGRP to bacteria

A range of model facultatively anaerobic bacteria (*E. coli* and *Bacillus subtilis*; Blades Biological Ltd, UK) and strict rumen anaerobes (*Pseudobutyrivibrio ruminis*, *Butyrivibrio spp., S. lutetiensis* and *Propionibacterium australiense*) were used to assess the binding activity of the recombinant *C. daubneyi* PGRP. The ruminal bacteria were isolated via the dilution-to-extinction method [135] in an anaerobic cabinet (Don Whitley Scientific, UK) using ruminal fluid from lambs fed a 50:50 grass silage-to-concentrate diet. Bacterial cultures (200 µl) in the mid-log phase (OD_600_ ~ 0.6) were mixed with recombinant PGRP (0–2 µg) and incubated for different time points at room temperature (18–21 °C). The cells were harvested by centrifugation at 5000 rpm for 10 min and washed three times in PBS. The final cell pellets were resuspended in LDS loading buffer and run on reducing 4–12% NuPAGE Bis–Tris gels (Life Technologies) and transferred to nitrocellulose membranes (GE Healthcare). Blots were probed with 1.0 µg/ml of an affinity-purified peptide antibody raised in rabbits against an antigenic peptide (CEQWGAQAPRTAHTK) of *C. daubneyi* PGRP (Genscript, NJ, USA) overnight at 4 °C. After washing, an appropriate alkaline phosphatase-conjugated IgG secondary antibody was applied to the membranes for 1 h at room temperature before detection using the BCIP/NBT substrate (Sigma-Aldrich).

### Peptidoglycan binding assay

Microtiter plates (96 well; Nunc) were coated with peptidoglycan from *Staphylococcus aureus* (10 µg/well; InvivoGen, USA) overnight at 4 °C. After blocking excess binding sites with 1% BSA (w/v) in PBS, recombinant *C. daubneyi* PGRP (0–5 µg/well) were added to the plate which was then incubated for 15 min at room temperature (18–21 °C). Binding of PGRP to peptidoglycan was detected by the addition of affinity-purified rabbit anti-CdPGRP (2 µg/ml in PBS) for 1 h at 37 °C, followed by alkaline phosphatase-conjugated goat anti-rabbit IgG (Sigma; 1:10,000 dilution PBS). Binding of secondary antibody was visualised by the addition of *p*-nitrophenol phosphate (Sigma; 80 µl/well) and measuring the absorbance at 405 nm. Binding specificity was demonstrated by adding recombinant PGRP to peptidoglycan-coated/uncoated plates or by adding trypsinised PGRP or a His-tagged recombinant *F. hepatica* thioredoxin glutathione reductase (TGR; R. Morphew) to the plates. TGR was detected by the addition of rabbit anti-FhTGR antibody (1:2000 dilution) as described above.

### Minimum inhibitory concentration

Minimum inhibitory concentration (MIC) assays for antimicrobials were performed according to the standard broth microdilution method [136], with minor modifications as described below. The assays utilised 96-well microtiter plates (Nunclon Delta-Treated, Flat-Bottom Microplate, ThermoFisher, UK) and were conducted in a Whitley A35 Workstation anaerobic chamber to ensure strict anaerobic conditions. *S. lutetiensis*, isolated from ovine ruminal fluid, was cultured without agitation overnight in Hungate tubes using a basal medium supplemented with 4 g/L glucose [137] at 39 °C. The bacterial inoculum was adjusted to approximately 1 × 10^5^ CFU/mL. Gentamicin sulphate (potency: ≥ 590 IU, Sigma-Alrich, UK) was prepared according to the manufacturer’s instructions and used as positive control. MIC values were assessed using 12 two-fold serial dilutions, starting at 0.025 mg/ml for gentamicin. The recombinant *C. daubneyi* PGRP was tested with and without 5 µM ZnSO_4_ (Sigma-Aldrich, UK) with a maximum concentration of 0.94 mg/ml. Each MIC experiment was conducted with two biological replicates, each comprising three technical replicates. The 96-well plates were incubated at 39 °C for 24 h. Post-incubation, bacterial viability was determined by the addition of 30 µL of a 0.1% (w/v) sterile resazurin solution (Sigma-Aldrich, UK) to each well, followed by an additional 30-min incubation under the same conditions [138]. The MIC was defined as the lowest concentration of antimicrobial at which the resazurin remained purple, indicating it was not reduced, thus signifying the inhibition of bacterial growth.

### Bacterial agglutination assay

*E. coli* and *B. subtilis* cultures in the mid-log phase (OD_600_ ~ 0.6) were collected by centrifugation (2500 × *g* for 8 min) and stained for 2 min in acridine orange (1% w/v; Sigma-Alrich, UK). After three washes with PBS, the cells were incubated in a 96-well plate with recombinant *C. daubneyi* PGRP (200 µg/ml) with and without 5 µM ZnSO_4_ for 1 h at room temperature. The cells, in biological triplicate, were observed using an EVOS Floid imaging system (ThermoFisher, UK).

### Isolation and extraction of fluke eggshells

Whole *C. daubneyi* and *F. hepatica* eggs were collected following in vitro culture of adult flukes in RPMI-1640 medium containing 0.1% w/v glucose, 100 U penicillin and 100 μg/ml streptomycin (Sigma-Aldrich), at 1 worm/ml for 5 h at 37 °C. Eggs were washed three times with PBS and processed to produce pure eggshell fragments using the method described by Waite and Rice-Ficht [87]. Briefly, approximately 250 µl of *C. daubneyi* or *F. hepatica* eggs (packed volume) were homogenised in 5% acetic acid with 8 M urea and incubated with gentle rotation for 6 h at room temperature. The sample was spun at 2000 × *g* for 5 min and the pellet was resuspended in 5% v/v acetic with 2 M urea and 0.01% w/v pepsin (Sigma-Alrich, UK) and digested overnight. Insoluble residue was collected by centrifugation as above and washed twice with 0.1 M borate buffer (pH 8.0) containing 2 M urea. Trypsin (0.01% w/v) was added and digestion was carried out overnight at room temperature. The final insoluble pellet, containing eggshell fragments, was collected by centrifugation and washed four times with deionised water.

### Mass spectrometry analysis of fluke eggshells

Purified eggshell fragments from *C. daubneyi* and *F. hepatica* (in biological triplicate) were solubilised in NuPage LDS loading buffer with 50 mM dithiothreitol as reducing agent (Life Technologies, UK) at 95 °C for 5 min and run on 4–12% NuPAGE Bis–Tris gels (Life Technologies). Gels were lightly stained with coomassie blue, destained with 45% v/v methanol and 10% v/v acetic acid and the dominant ~ 29 kDa band was excised for mass spectrometry analysis. The gel pieces were digested with 100 ng/μl sequencing grade trypsin (Promega, UK) overnight at 37 °C. Tryptic peptides were dried in a vacuum centrifuge and reconstituted with 10 µL of 0.1% TFA before analysis by liquid chromatography-tandem mass spectrometry (LC–MS/MS) using a LTQ OrbiTrap Velos Pro (Thermo Scientific) as previously described [23].

All MS/MS samples were analysed using MASCOT (Matrix Science, London, UK; version 2.6.2). MASCOT was set up to search the *C. daubneyi* predicted proteome database (version 1.0, 13,292 entries) assuming the digestion enzyme strict trypsin. MASCOT was searched with a fragment ion mass tolerance of 0.060 Da and a parent ion tolerance of 10.0 PPM. Carbamidomethyl of cysteine was specified as a fixed modification whilst Gln- > pyro-Glu of the N-terminus, deamidation of asparagine and glutamine, oxidation of methionine, dioxidation of methionine and acetyl of the n-terminus were specified as variable modifications. Scaffold (version Scaffold_4.4.5, Proteome Software Inc., Portland, OR, USA) was used to validate MS/MS-based peptide and protein identifications. Peptide identifications were accepted if they could be established at greater than 95.0% probability by the Scaffold Local FDR algorithm. Protein identifications were accepted if they could be established at greater than 99.0% probability and contained at least 2 identified peptides. Protein probabilities were assigned by the Protein Prophet algorithm [139]. Proteins that contained similar peptides and could not be differentiated based on MS/MS analysis alone were grouped to satisfy the principles of parsimony. Proteins sharing significant peptide evidence were grouped into clusters. Additionally, a label-free quantitative analysis (based on the exponentially modified protein abundance index; emPAI) performed in Scaffold was used as a measure of protein abundance. Only proteins with at least two unique peptides, that were present in all three biological replicates, were retained.

### Amino acid analysis of fluke eggshells

Whole eggs and eggshell fragments were hydrolysed under vacuum in sealed ampules with 100 µl of 6 M HCl and 5 µl of phenol for 24 h at 110 °C. The resulting amino acid constituents were analysed with a Hitachi L-8900 Amino Acid Analyzer calibrated with an amino acid standard solution (Sigma-Aldrich) and 3,4-dihydroxyphenylalanine (DOPA; Sigma-Aldrich). Each sample was run in triplicate.

### Egg stability assays

Approximately 200 *C. daubneyi* and *F. hepatica* eggs were suspended in increasing sodium hypochlorite concentrations (0–1%) and incubated, in biological triplicate, for 1 h at room temperature (18–21 °C). Eggs were similarly incubated in buffers with varying pH: 0.1 M sodium acetate (pH 4.0), PBS (pH 7.0) or 0.1 M sodium borate (pH 9.0) at room temperature and observed at 0-, 1- and 4-h incubations according to Afifi and Ali [51].

### Histochemistry and electron microscopy

For histological examination, adult *C. daubneyi* were immediately fixed in 10% w/v neutral buffered formalin for 24 h after their removal from the rumen according to Hanna et al. [140]. Flukes were then dehydrated in ethanol, cleared in Clearene (Surgipath, UK), and embedded in wax following conventional procedures. Sections (5 µm) were cut, stained with haematoxylin and eosin or Mallory’s Trichrome (Sigma-Aldrich, UK) and viewed with a Leica DM LN2 microscope with a Nikon Coolpix 5000 camera system. Whole mounted flukes were stained in 0.1% w/v catechol (Sigma-Aldrich, UK) as previously described [49].

For transmission electron microscopy (TEM), adult *C. daubneyi* were fixed in 4% w/v glutaraldehyde in 0.1 M sodium cacodylate buffer (pH 7.4) containing 3% w/v sucrose for 1 h at room temperature. After fixation, the material was washed in 0.1 M sodium cacodylate buffer (pH 7.4) containing 3% w/v sucrose overnight. This was followed by post-fixation for 1 h in 1% v/v aqueous osmium tetroxide (Agar scientific, UK), dehydration through an ethanol series and embedding in Agar 100 epoxy resin. Ultrathin sections were mounted on uncoated copper grids, double-stained with 1% w/v uranyl acetate and 1% w/v lead citrate, and viewed using a JEOL JEM-1400 transmission electron microscope with an AMT Activue XR16 digital camera system, operating at an accelerating voltage of 80 kV.

## Supplementary Information

Additional file 1. Conservation of KEGG modules across selected platyhelminth species. Raw data (gene counts/proportion of complete modules) used to generate the heatmap shown in Fig. 2.

Additional file 2. Characteristics of the *C. daubneyi* PGRP family. Features of the 23 *C, daubneyi* PGRPs including amino acid (AA) sequence length, molecular mass (kDa), classification of long (L) and short (S) forms, conserved domains detected by InterPro and presence of transmembrane regions and signal peptide for secretion are shown.

Additional file 3.Phylogenetic analysis of the *C. daubneyi* PGRP, SAPLIP and DM9CP families. Neighbour-joining trees showing the evolutionary histories of the *C. daubneyi* PGRP, SAPLIP and DM9CP families. Bootstrap values (1000 replicates) are shown as percentages for a particular node. The major clades/sub-clades for each family are numbered.

Additional file 4. Expression and purification of a recombinant *C. daubneyi* PGRP in *E. coli*. Full-length *C. daubneyi* PGRP was expressed as a recombinant protein bearing a C-terminal His_6_-tag in *E. coli*. The protein was purified from *E. coli* cell extracts using Ni-affinity chromatography and run on a reducing SDS-PAGE gel stained with Coomassie blue (1). Western blot (2) showing the purified recombinant protein probed with an anti-His antibody. M, molecular weight marker.

Additional file 5. Comparative histochemical analysis of *C. daubneyi* and *F. hepatica*. (A) Tissue sections showing differential staining of adult *F. hepatica* (Fh) and *C. daubneyi* (Cd) with Mallory’s Trichrome. In *F. hepatica* the eggshell protein globule clusters within the vitelline follicles, the eggshell of fully formed eggs within the uterus, and those shed by the flukes in vitro, appeared golden brown whilst the equivalent structures in *C. daubneyi *stained a deep red colour. Whole mount preparation of adult *F. hepatica* (B) stained with catechol. The vitelline follicles (arrowed) extend throughout the lateral margins of the fluke and stain golden brown, indicative of tyrosinase. In contrast, in catechol-stained adult *C. daubneyi* (C) no staining is evident.

Additional file 6. Amino acid analysis of *C. daubneyi* and *F. hepatica* eggshells. Whole eggs and eggshell fragments were subjected to acid hydrolysis and the resulting amino acid constituents analysed with a Hitachi L-8900 Amino Acid Analyzer against an internal amino acid reference standard. Each sample was run in triplicate.

Additional file 7. Proteomics analysis of *C. daubneyi* and *F. hepatica* eggshells. Identification of *C. daubneyi* and *F. hepatica* eggshell proteins by LC-MS/MS. Samples were run in biological triplicate and only proteins with at least two unique peptides, that were present in all three biological replicates, were retained.

Additional file 8. Egg stability assays. Whole eggs were recovered from adult *F. hepatica* (Fh) and *C. daubneyi* (Cd) following in vitro culture and incubated for 4 h in buffers of different pH (A) or in increasing sodium hypochlorite concentrations for 1 h (B) at room temperature. The integrity of the eggshells was monitored by light microscopy. Images are representative of triplicate experiments.

Additional file 9. Extracellular digestion of ciliates in the *C. daubneyi* gut. (A) Haematoxylin and eosin-stained tissue section of adult *C. daubneyi* showing ciliates (arrowed) and in close up (B and C) within the oral cavity/foregut. The recognisable structure of the ciliates (*) becomes lost as these move deeper within the gut caeca and begin to break down (D). Os, oral sucker; Gc, gastrodermal cells.

Additional file 10. Single copy orthologous groups used for phylogenetic analysis. Orthologous groups identified via Orthofinder analysis, of which 180 shared single copy orthologues from *C. daubneyi* and 17 other flatworm species, representing the major taxonomic groups were used for phylogenetic analysis.

Additional file 11. Unprocessed blots shown in Fig. 5A-C.

## Data Availability

The datasets supporting the conclusions of this article are available from the European Nucleotide Archive under accessions PRJEB76148 [141] (genomic read data) and PRJEB28150 [23, 142] (transcriptomic read data). The MS proteomics data have been deposited to the ProteomeXchange Consortium via the PRIDE partner repository with the dataset identifier PXD053790 [143]. Unprocessed blots are provided in Additional File 11.
