# Supplementary figures and images for: The Calicophoron daubneyi genome provides new insight into mechanisms of feeding, eggshell synthesis and parasite-microbe interactions
Source: BMC Biol. 2025 Jan 13;23:11. doi: 10.1186/s12915-025-02114-0 (PMC11727788; doi:10.1186/s12915-025-02114-0)

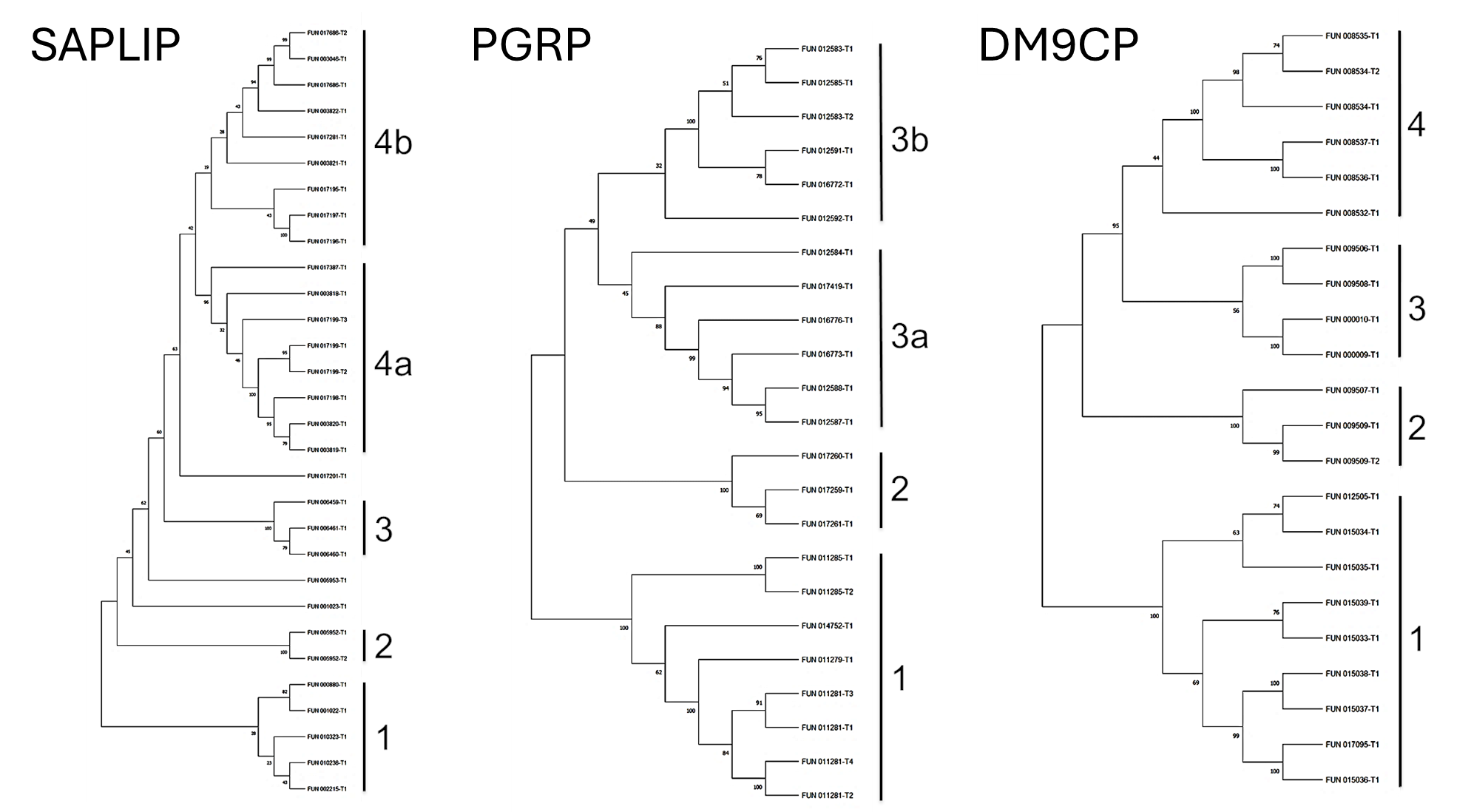

Supplement: Supplementary file 3 — Additional file 3.Phylogenetic analysis of the C. daubneyi PGRP, SAPLIP and DM9CP families. Neighbour-joining trees showing the evolutionary histories of the C. daubneyi PGRP, SAPLIP and DM9CP families. Bootstrap values (1000 replicates) are shown as percentages for a particular node. The major clades/sub-clades for each family are numbered. [file 12915_2025_2114_MOESM3_ESM.tif]

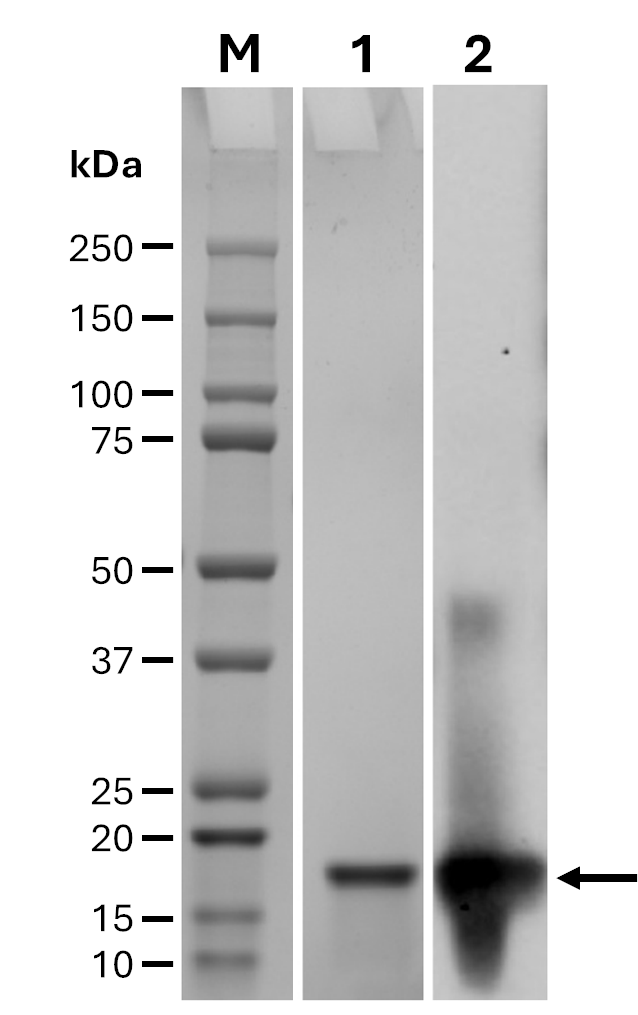

Supplement: Supplementary file 4 — Additional file 4. Expression and purification of a recombinant C. daubneyi PGRP in E. coli. Full-length C. daubneyi PGRP was expressed as a recombinant protein bearing a C-terminal His6-tag in E. coli. The protein was purified from E. coli cell extracts using Ni-affinity chromatography and run on a reducing SDS-PAGE gel stained with Coomassie blue (1). Western blot (2) showing the purified recombinant protein probed with an anti-His antibody. M, molecular weight marker. [file 12915_2025_2114_MOESM4_ESM.tif]

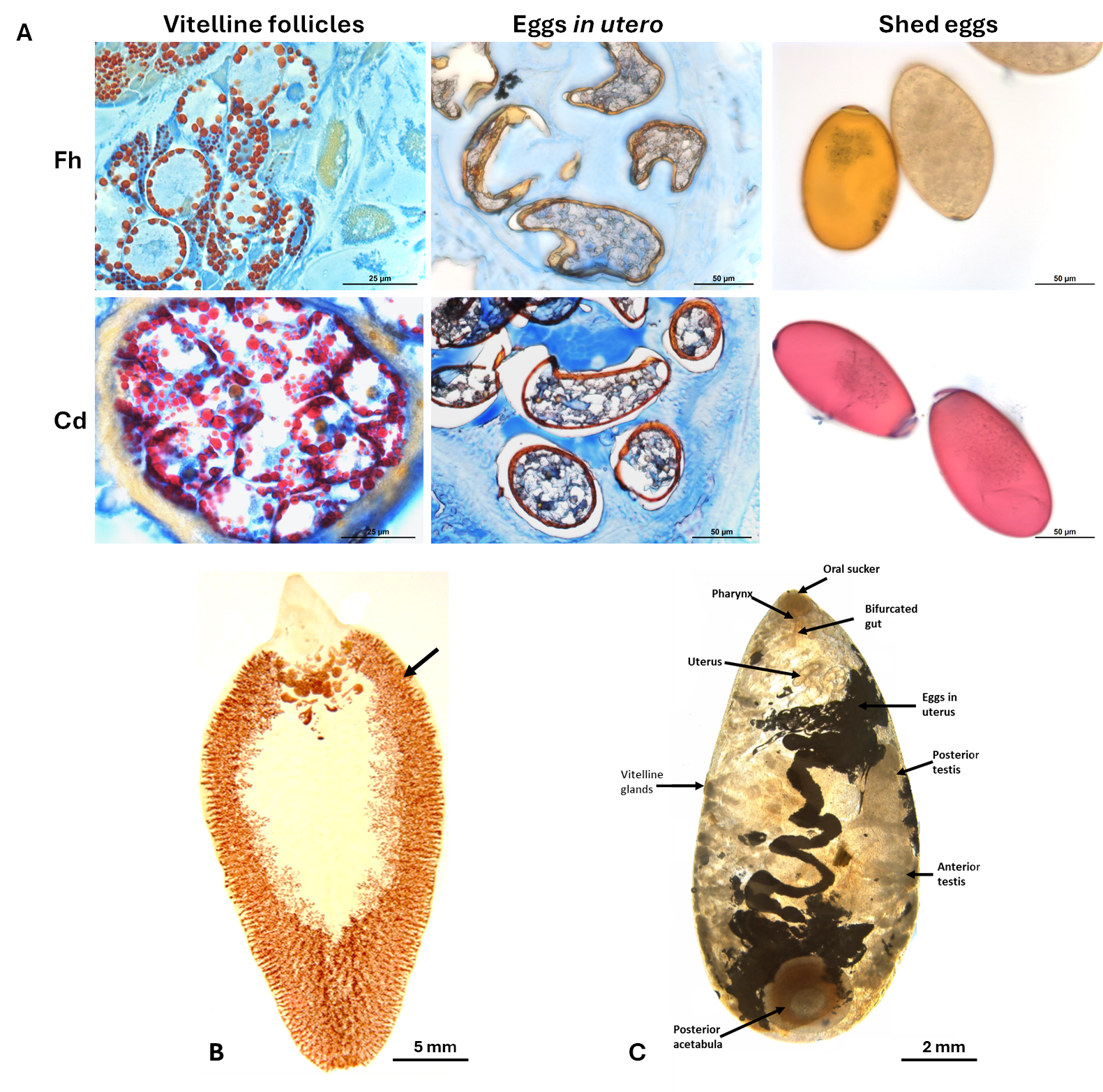

Supplement: Supplementary file 5 — Additional file 5. Comparative histochemical analysis of C. daubneyi and F. hepatica. (A) Tissue sections showing differential staining of adult F. hepatica (Fh) and C. daubneyi (Cd) with Mallory’s Trichrome. In F. hepatica the eggshell protein globule clusters within the vitelline follicles, the eggshell of fully formed eggs within the uterus, and those shed by the flukes in vitro, appeared golden brown whilst the equivalent structures in C. daubneyi stained a deep red colour. Whole mount preparation of adult F. hepatica (B) stained with catechol. The vitelline follicles (arrowed) extend throughout the lateral margins of the fluke and stain golden brown, indicative of tyrosinase. In contrast, in catechol-stained adult C. daubneyi (C) no staining is evident. [file 12915_2025_2114_MOESM5_ESM.tif]

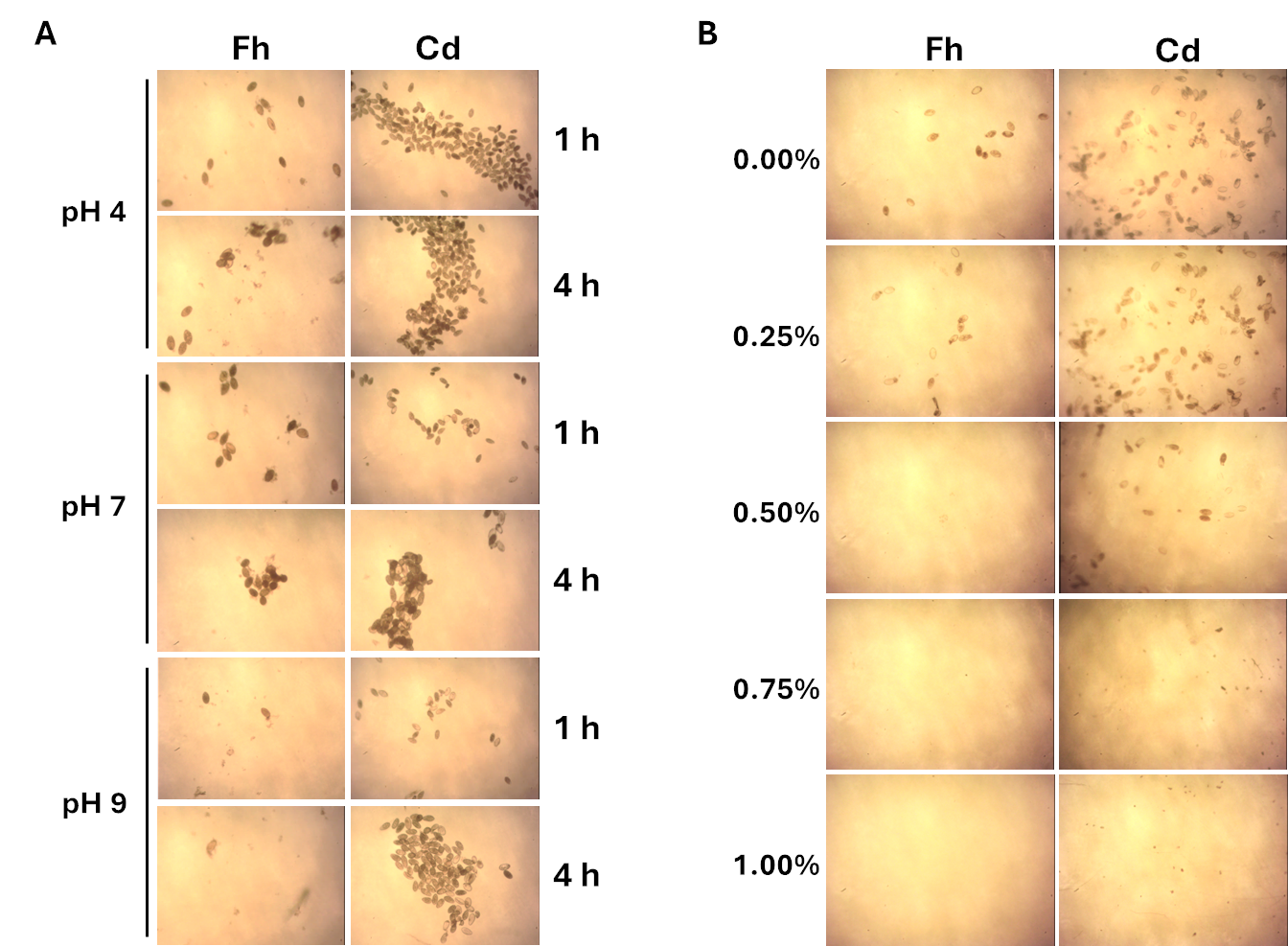

Supplement: Supplementary file 8 — Additional file 8. Egg stability assays. Whole eggs were recovered from adult F. hepatica (Fh) and C. daubneyi (Cd) following in vitro culture and incubated for 4 h in buffers of different pH (A) or in increasing sodium hypochlorite concentrations for 1 h (B) at room temperature. The integrity of the eggshells was monitored by light microscopy. Images are representative of triplicate experiments. [file 12915_2025_2114_MOESM8_ESM.tif]

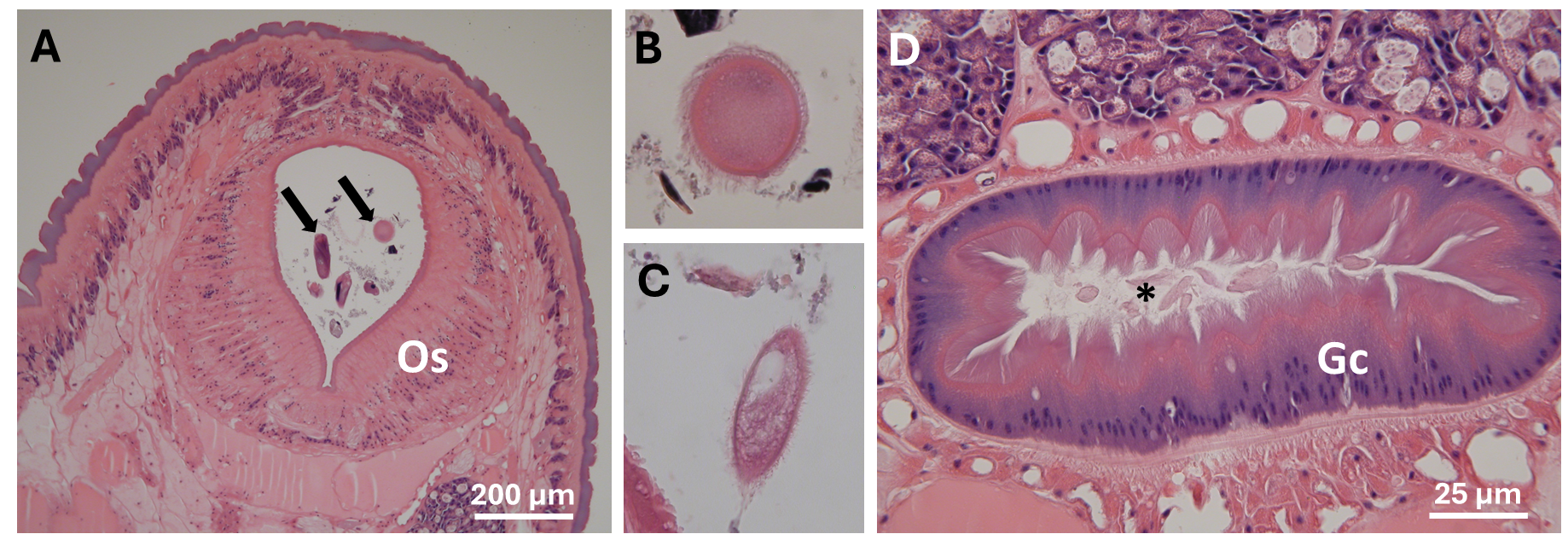

Supplement: Supplementary file 9 — Additional file 9. Extracellular digestion of ciliates in the C. daubneyi gut. (A) Haematoxylin and eosin-stained tissue section of adult C. daubneyi showing ciliates (arrowed) and in close up (B and C) within the oral cavity/foregut. The recognisable structure of the ciliates (*) becomes lost as these move deeper within the gut caeca and begin to break down (D). Os, oral sucker; Gc, gastrodermal cells. [file 12915_2025_2114_MOESM9_ESM.tif]

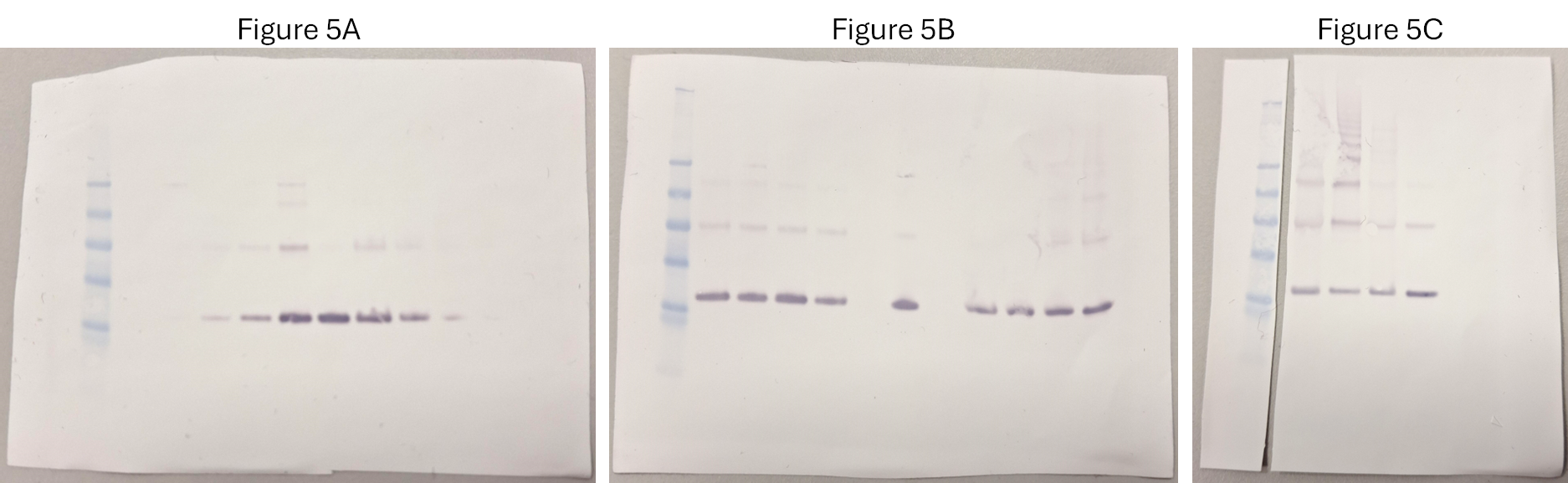

Supplement: Supplementary file 11 — Additional file 11. Unprocessed blots shown in Fig. 5A-C. [file 12915_2025_2114_MOESM11_ESM.tif]
